# Modulation of biological activities in adipose derived stem cells by histone deacetylation

**DOI:** 10.1038/s41598-024-84652-1

**Published:** 2025-01-29

**Authors:** Sallam Abdallah, Mouna Tabebi, Sawsan Qanadilo, Neserin Ali, Jing Wang, Pádraig D’Arcy, Wen Zhong, Folke Sjoberg, Moustafa Elmasry, Ahmed El-Serafi

**Affiliations:** 1 The Department of Biomedical and Clinical Sciences (BKV), Linköping University, Linköping, Sweden; 2 Department of Biological Sciences, The University of Jordan, Amman, Jordan; 3 Department of Clinical Sciences, Lund University, Lund, Sweden; 4 Department of Hand Surgery and Plastic Surgery and Burns, University Hospital, Linköping, Sweden; 5 Clinical Genomics Linköping, Linköping University, Linköping, Sweden

**Keywords:** Histone deacetylase inhibitor, Suberoylanilide hydroxamic acid, Adipose derived stem cells, Differentiation, Vorinostat, Epigenetic, Epigenetic modifier, Cell biology, Molecular biology, Stem cells

## Abstract

Difficult-to-heal wounds management accounts for about 4% of healthcare costs, highlighting the need for innovative solutions. Extracellular signals drive cell proliferation during tissue regeneration, while epigenetic mechanisms regulate stem cell homeostasis, differentiation, and skin repair. Exploring epigenetic regulation in adipose-derived stem cells (ADSCs) holds promise for improving skin injury treatments. We investigated the effects of histone deacetylase inhibitor (SAHA) on ADSCs to better understand its cellular and molecular impacts. ADSCs were treated with SAHA for 72 h, showing no change in cell viability at the studied concentrations. However, the expression of histone deacetylase decreased at 1000 nM, while the cell proliferation marker Ki-67 increased after SAHA treatment, as confirmed by immunofluorescence. CCND1 gene expression increased, whereas protein expression of the proliferating cell nuclear antigen (PCNA) decreased. Cell cycle analysis showed an increase in G2 phase in SAHA-treated cells. Microarray analysis revealed 74 upregulated and 40 downregulated differentially expressed genes, including upregulation of P53 targets, *CDKN1A* and *MDM2*. Proteomic analysis identified 631 upregulated and 823 downregulated proteins compared to the vehicle. Pathway enrichment analysis showed cell cycle, ATP-dependent chromatin remodeling and DNA processes were among the affected pathways. This study suggests SAHA modulates ADSCs’ biological processes, highlighting its potential for skin regeneration.

## Introduction

The consistent exposure of the skin to environmental, mechanical, and chemical stress necessitates continuous self-renewal, in order to retain its barrier function. Skin has an extremely fast cellular turnover rate, which allows for efficient adaption to environmental triggers^1^. Skin cells response for the latter encompasses several epigenetic mechanisms for controlling gene expression which, in turn, modifies cellular phenotype, proliferation, migration, and differentiation^2^. Histone acetylation is one of the well-explained post-translational modifications, which is based upon the opposing activities of histone acetyltransferase (HATs) and histone deacetylase (HDAC)^3^. The importance of histone acetylation has been demonstrated in skin development and homeostasis, paving the way for potential investigations into its role during the repair of skin injuries^2^. For example, the acetylation pattern of epidermal histone H4 varied over time, during different stages of wound healing in distant and adjacent skin, migrating epithelium and newly formed epidermis^4^. Furthermore, epigenetic mechanisms control the differentiation and commitment outcomes of epidermal stem cells in wound healing process as the restoration of functional skin relies on the plasticity of stem cells^5^. Epigenetic modification was identified as a fundamental contributor to stem cell-mediated tissue maintenance, by coordinating their fate decisions and safeguarding their homeostasis^6^. The epigenetic profiles are emerging for skin stem cells that are associated with their cell fate plasticity and proper activity in tissue regeneration, the role and the mechanism of which adipose derived stem cells use to contribute to restoration of skin disruption, is largely unclear.

Adult stem cells stand out as a preferred choice for stem cells based therapy with increasing body of evidence confirming their safety, as well as their efficacy in regeneration and repair^7^. Most attention is drawn to adipose derived stem cells (ADSCs), as a special subset of adult stem cells with the advantage of their abundance and ease of access, in addition to their ability to self-renew, differentiate into multilineages, and dynamically interact with the cell niche^8^. On the other hand, ADSCs are a source of bioactive growth factors which participate in paracrine signaling during tissue repair process, including skin wound healing. The interplay between ADSCs and the surrounding environment can provide important aspects for improving their use in clinical practice. They may participate in wound repair and reconstruction as well as the inflammatory response^9^.

Epigenetic pharmaceuticals are promising interventions as they support stem-cell differentiation^10–15^. Histone deacetylase inhibitors (HDACi) are known to modulate the expression of genes by increasing histone acetylation and thereby regulating chromatin structure and transcription^16^. Current evidence indicates that HDAC inhibitors not only act to reduce the catalytic activity of the enzyme but may also disrupt the protein–protein interaction of specific HDACs with various critical protein partners^17^. These target proteins usually participate in a variety of biological pathways, including gene expression, cell proliferation, differentiation, cell migration, and cell death, as well as angiogenesis and immune response^18^. Therefore, we opted to use pharmacological manipulation to regulate histone acetylation by using HDACi, Suberoylanilide hydroxamic acid (SAHA), also known as Vorinostat. This pan HDACi inhibits both the enzymes from class I (HDAC1, HDAC2, HDAC3) and class II (HDAC4, HDAC5, HDAC6 and HDAC7). Although several HDACi are at various stages of preclinical and clinical development, SAHA, is a clinically approved histone deacetylase inhibitor, by the U.S. Food and Drug Administration (FDA) for the treatment of T-cell lymphomas^19^.

In this study, we hypothesize that altering ADSCs through pharmacological intervention with SAHA may represent a potential therapeutic strategy to improve wound therapy and skin regeneration. To test this, we investigated the effect of SAHA on ADSCs proliferation and the regulatory pathways involved in various biological processes, aiming at enhancing the application of ADSCs in cell therapy for skin tissue regeneration. To the best of our knowledge, no studies have examined the effect of SAHA on ADSCs cellular and molecular processes. Therefore, our data could provide valuable insights for developing novel therapeutic approaches to treat sever skin injuries.

## Results

### The reduction of HDACs demonstrates no adverse impact on cell viability

**Fig. 1:**
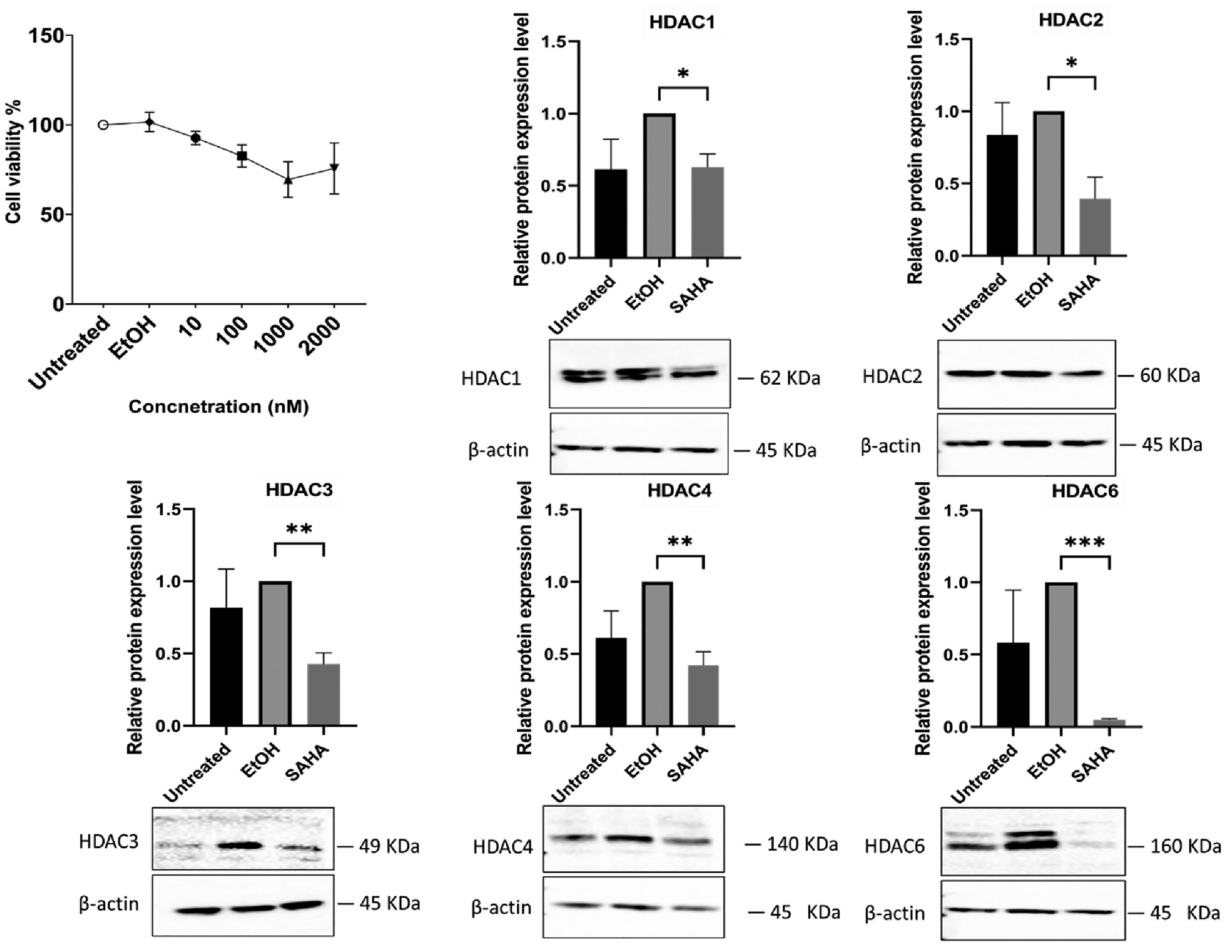
The viability of ADSCs remains unaffected by SAHA despite the significant reduction in the protein expression levels of HDACs. (**A**) ADSCs viability percentages were assessed through the MTT assay after a 72-h with different concentrations of SAHA (10, 100, 1000, and 2000 nM). The control group for this experiment was untreated cells. (**B**) Western blotting was performed to analyze HDACs protein expression level and showed significant reduction of HDAC1,2,3,4 and 6. Data were presented as means with the corresponding standard error of the mean (SEM), the original blots/gels can be accessed in Supplemetary 1. MTT assay (n = 4) and for western blotting (n = 3). *p* value < 0.05 was indicating of a statistically significant difference, which presented as follows: * for *p* < 0.05, ** for *p* < 0.01, and *** for *p* < 0.001.

The viability of ADSCs were examined after being treated with SAHA at different concentrations (Fig. 1A). Our findings revealed no changes in viability with the studied concentrations, in comparison to untreated cells. In the following experiments, 1000 nM SAHA was used at 72 h of exposure. To determine whether SAHA has efficiently inhibited HDACs at 1000 nM, the protein expression of certain HDACs was screened. HDACs expression level in SAHA group were significantly reduced for HDAC1, HDAC2, HDAC3, HDAC4 and HDAC6 in comparison with EtOH group (Fig. 1B). This can demonstrate that the method of application used in our study positively influenced the reduction of HDACs level.

### ADSCs displayed active division despite evidence of slow proliferation rate

**Fig. 2:**
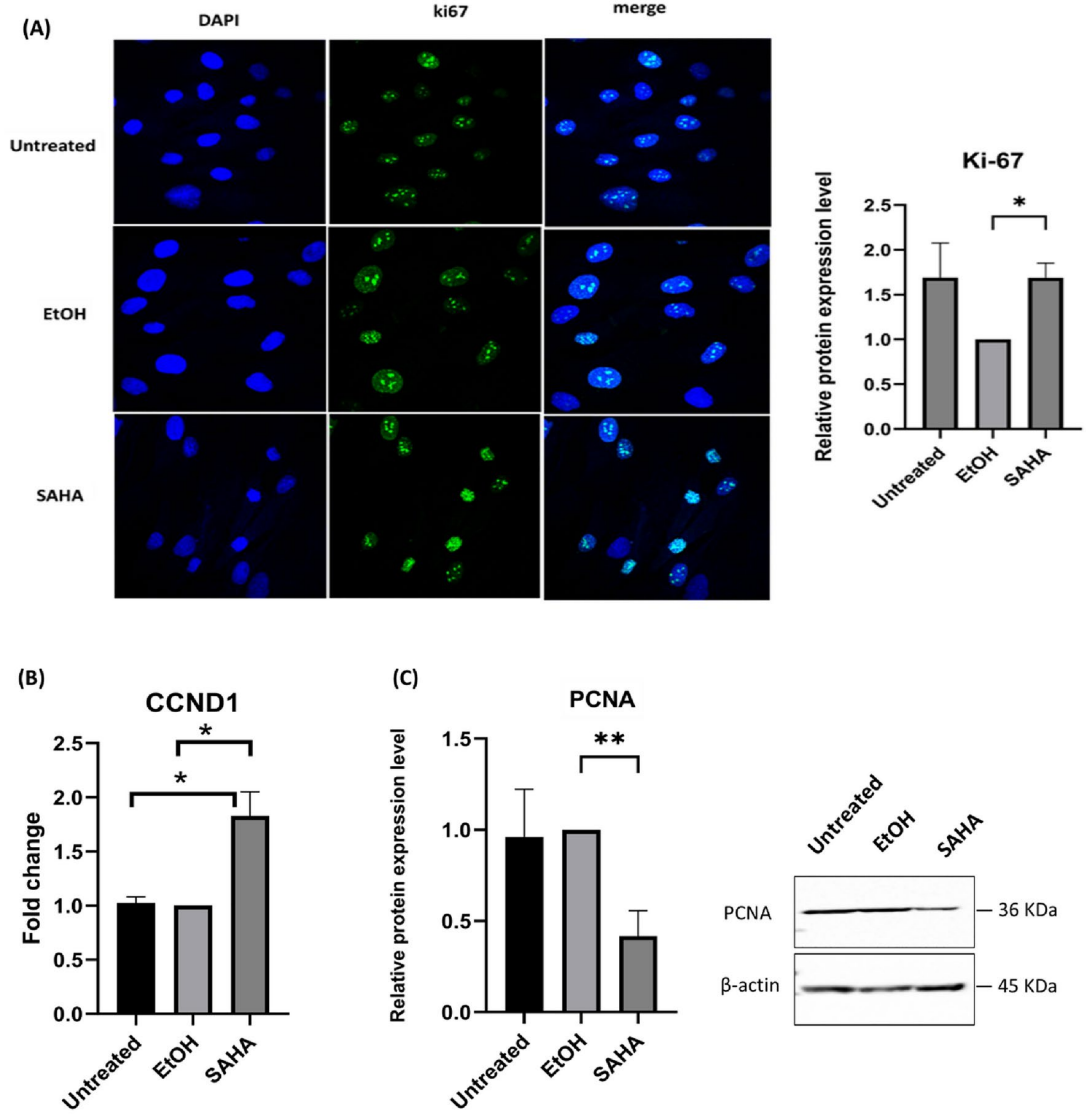
Proliferation capability of ADSCs under the effect of SAHA**. **(**A**) Quantification of Ki-67 fluorescence intensity in ADSCs was determined using immunofluorescence staining. (**B**) CCND1 gene expression using qPCR. (**C**) PCNA relative protein expression was evaluated by western blotting, the original blots/gels can be accessed in Full-length blots file. Data were presented as means with the corresponding standard error of the mean (SEM). qPCR (n = 3) and western blotting (n = 4). *p* value < 0.05 was indicating of a statistically significant difference, which presented as follows: * for *p* < 0.05, ** for *p* < 0.01, and *** for *p* < 0.001.

ASC52 cell cycle progression was performed by measuring DNA content which was stained with propidium iodide (PI) using flowcytometry. The stained DNA histogram, presented in Supplementary 2 Fig. 2), along with the quantification of DNA content percentages. Our results demonstrated an increase in G2/M cell cycle phases with no increase in Sub G1 population in SAHA treated cells. Following treatment with SAHA, we observed notable changes in the morphological features of the cells compared to untreated controls. The cells exhibited a polygonal shape with a larger cytoplasm, which was clearly visible under the light microscope (Supplementary 2 Fig. 1). These morphological alterations suggest changes at the cellular levels. As these changes could be related to cell proliferation, we investigated the expression of the Ki-67 protein. Our results revealed a significant increase in Ki-67 protein expression in SAHA group compared with EtOH group. Nevertheless, the expression level was similar to the control group (Fig. 2A). For a better understanding of SAHA influence on ADSCs cell cycle progression and proliferation, the gene expression of the cell cycle G1/S transition check point *CCND1* was investigated. A significant increase in the expression of *CCND1* gene expression was observed in SAHA group compared with EtOH group (Fig. 2B). Furthermore, the nuclear protein PCNA, a cellular proliferation marker, was studied and a significant decrease was observed compared to the EtOH group (Fig. 2C).

### Microarray analysis identification of genes and pathways on ADSCs

**Fig. 3:**
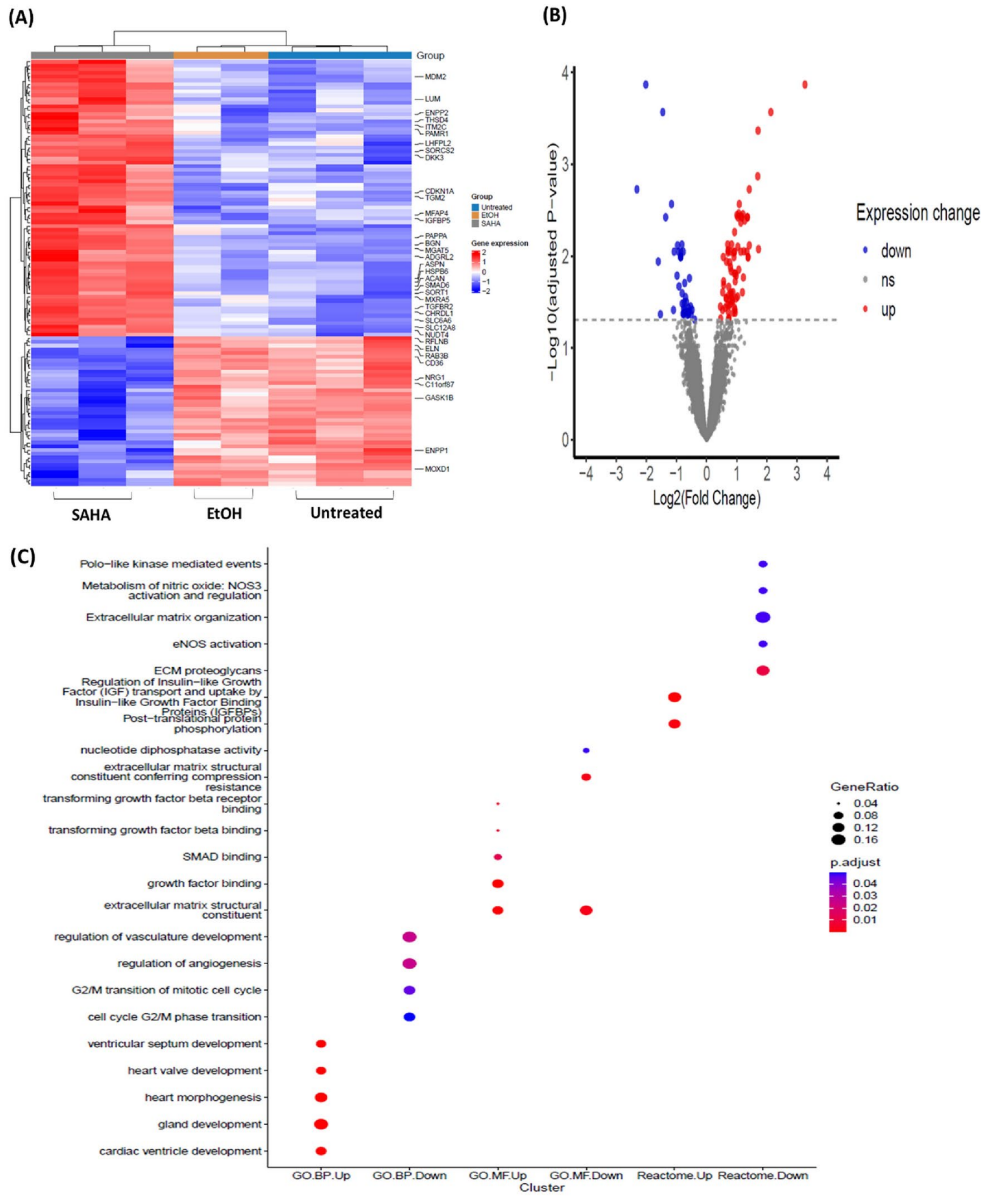
Microarray analysis for the differentially expressed genes and enrichment pathways analysis. (**A**) Heat map of the top110 genes and (**B**) Volcano plot for the differentially expressed genes. (**C**) Bubble plot shows the top enriched pathways of DEGs from Gene ontology and reactome. Data were presented as means with the corresponding standard error of the mean (SEM). n = 3. *p* value < 0.05 was indicating of a statistically significant difference, which presented as follows: * for *p* < 0.05, ** for *p* < 0.01, and *** for *p* < 0.001.

**Fig. 4:**
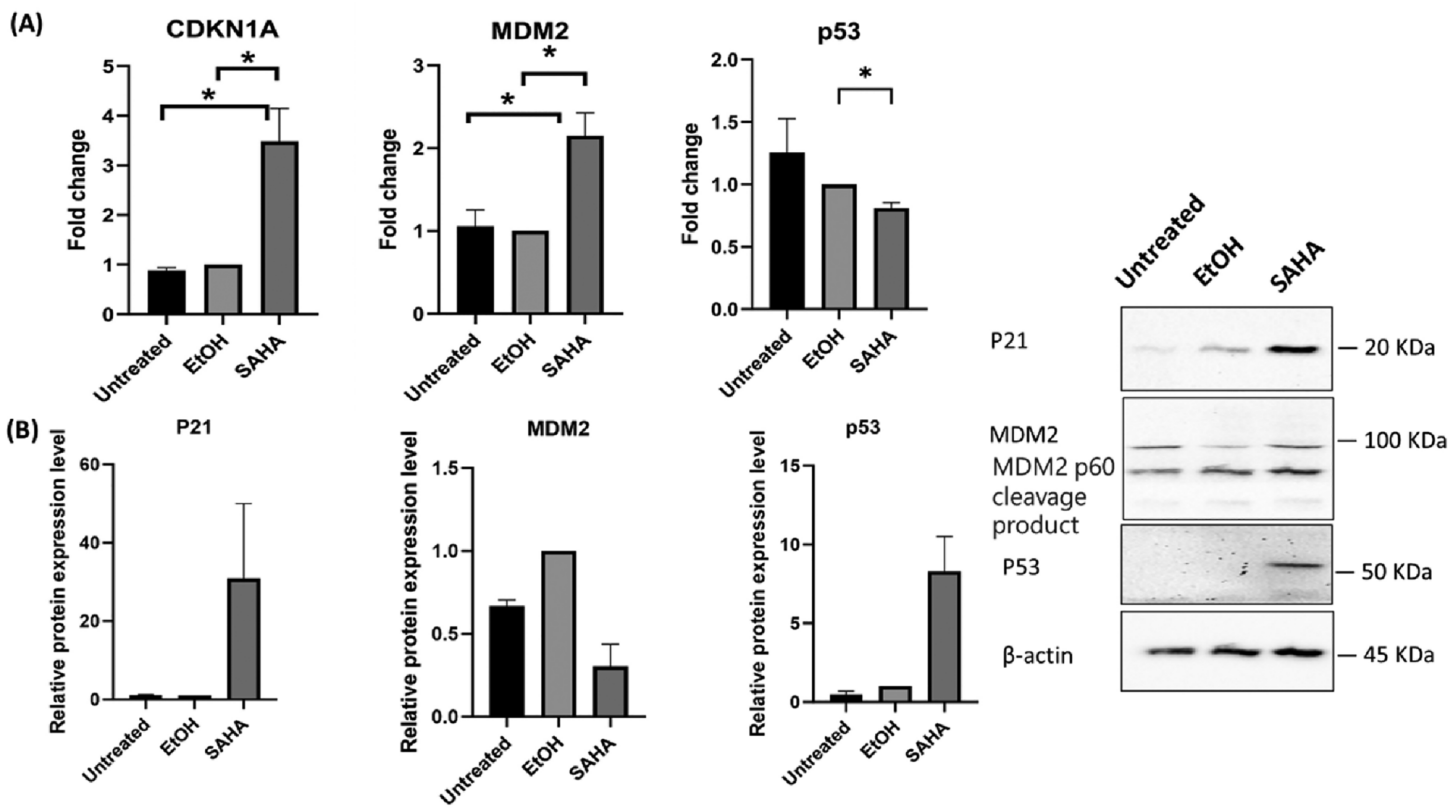
p21/p53 evaluation on ASC52 under the influence of SAHA treatment (A) Differentially expressed genes in relation to p53 pathway using qPCR. (B) Protein expression in relation to p53 target genes using western blotting, For MDM2 blot shows both full length and cleaved MDM2 fragment called p60. The original blots/gels can be accessed in Full-length blots file. Data were presented as means with the corresponding standard error of the mean (SEM). qPCR (n = 3), western blotting (n = 2). *p* value < 0.05 was indicating of a statistically significant difference, which is presented as follows: * for *p* < 0.05, ** for *p* < 0.01, and *** for *p* < 0.001.

To characterize the effect of SAHA on ADSCs biological activity, microarray analyses were performed to identify genes and regulatory pathways which influence the cellular and molecular mechanisms in ADSCs after SAHA treatment. Our findings showed that 40 genes were downregulated, while 70 genes were upregulated (Fig. 3A, B), (Supplementary 3). For the downregulated genes, the top five enriched pathways from reactome pathway analysis were related to extracellular matrix organization and metabolism of nitic oxide, while the upregulated genes were related to the regulation of insulin-like growth factor (IGF) transport and uptake by insulin-like growth factor binding proteins (Fig. 3C), (Supplementary 2 Table 2–5). To confirm microarray results, candidate genes from the previously mentioned enriched pathways were selected. The qPCR results confirmed the microarray data (Supplementary 2 Fig. 2), (Supplementary 2 Table 6–7). Moreover, the expression of p53/p21(CDKN1A) pathway related gene were among the influenced genes by SAHA. A significant increase in *CDKN1A* and *MDM2* gene expression was observed in response to SAHA treatment, while p53 gene expression exhibited a notable decrease (Fig. 4A). Notably, we observed similar trends in the protein levels of p21, which was accompanied with an increase in p53 and a decrease in MDM2. This indicates that SAHA activates a p53 response in the context of ADSCs, subsequently leading to the activation of certain p53 target genes (Fig. 4B).

### Proteomic profiling showed regulation of cell fate related pathways

**Fig. 5:**
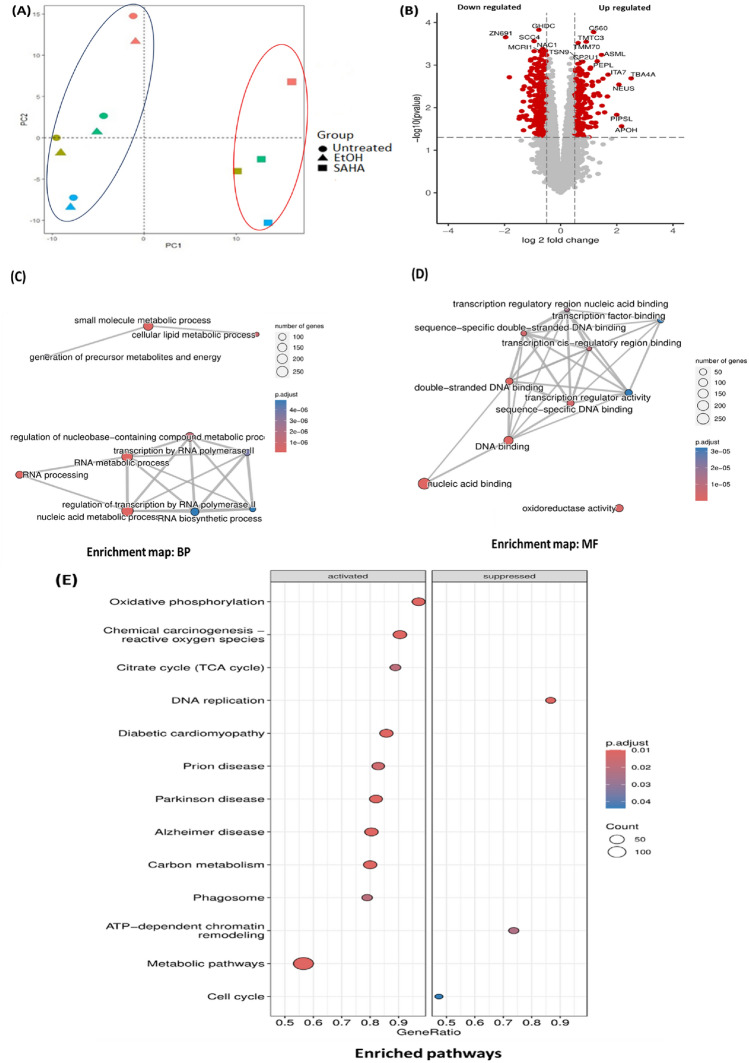
Differentially expressed proteins identification using mass spectrometry analysis identified; 823 proteins were downregulated while 631 were upregulated. (**A**) Principal components analysis (PCA) showed the distribution of the identified proteins among different groups. (**B**) Volcano plot for the downregulated and upregulated differentially expressed proteins which are shown in red, while the proteins in gray are non-significant. (**C, D**) Gene ontology enrichment maps for biological process and molecular function. (**E**) Bubble plot showing pathways enrichment analysis for the regulated downstream pathways. Data were presented as means with the corresponding standard error of the mean (SEM). n = 5. *p* value < 0.05 was indicating of a statistically significant difference, which presented as follows: * for *p* < 0.05, ** for *p* < 0.01, and *** for *p* < 0.001.

The proteomics profile of ADSCs lysates were screened using mass spectrometry coupled with independent acquisition data analysis (DIA). Principle component analysis (PCA) showed the overlapping clustering of EtOH and control group with distinct separation of the SAHA group (Fig. 5A). A pairwise analysis in SAHA vs EtOH group showed 1454 differentially expressed proteins; 823 proteins were downregulated while 631 were upregulated proteins (Fig. 5B), (Supplementary 4, 5). Gene ontology reflected the biological function of RNA and its metabolic processing, while most of the molecular function were related to DNA binding (Fig. 5C, D), (Supplementary 2 Table 8, 9). Pathway enrichment analysis showed that cell cycle, ATP-dependent chromatin remodeling and DNA replication are among the suppressed regulated pathways (Fig. 5E), (Supplementary 2 Table 10).

## Discussion

Epigenetics regulation is involved in several biological processes in skin repair, and dynamically regulates cell proliferation, differentiation, and migration. Epigenetic regulatory mechanisms can both stimulate and repress gene activation, which alter cellular phenotype and behavior^20^. Histone acetylation is a key epigenetic process that neutralizes the positive charge on the histone surface, leading to a less compact chromatin structure. In contrast, histone deacetylation generally results in a tighter chromatin structure, thereby repressing gene expression^21^. Epigenetic regulators, including histone deacetylases (HDACs), are well known to play a crucial role in various processes governing gene expression, including the regulation of epidermal stem cells^22^. Furthermore, modulation of epigenetic signals associated with alterations of lineage-specific differentiation, could aid in the creation of modified therapeutic approaches. ADSCs are among the types of stem cells sources which have received much attention due to lack of invasive characteristics and high self-renewal^9^. In our study, we focused on demonstrating the effect of HDACi, SAHA, as a pharmaco-epigenetic intervention on ADSCs. Drug concentration appears to be a critical factor in screening HDACi, as most HDACi exhibit cytotoxicity and their up-take ability is time and cell type dependent. Our results showed no difference in cell survival within the studied concentration of SAHA and 1000 nM was selected as this concentration has been used in similar studies^12,13^. HDACi can decrease the expression of certain HDAC proteins in stem cells, but the effect in this context varies and depends on cell type and the specific HDAC inhibitors used. In our study model, HDACs protein expression was noticeably decreased in ADSCs treated with SAHA. On the other hand, Paino et al*.* investigated the impact of valproic acid (VPA), another HDACi, on the differentiation of human dental pulp stem cells (DPSCs) into osteoblasts. Their findings showed that VPA does not generate toxic effects on DPSCs and is accompanied by an increase in overall histone acetylation^23^. In another study, low concentrations of MS-275 showed no significant effects on DPSC proliferation compared to the control group. However, the proliferation of DPSC decreased at higher concentrations of MS-275^24^. With this respect, the proliferation of ADSCs treated with SAHA was investigated using Ki67. Ki67 is present during active phases of the cell cycle and is absent in resting cells (G0 phase), with maximal Ki-67 levels detected during mitosis^25^. Our findings demonstrated a considerable increase of Ki-67 protein expression in ADSCs treated with SAHA in comparison to the EtOH group. This was in line with measurement of Ki-67 protein levels in human immortalized foreskin (hTERT-BJ), during quiescence and different stages of cell cycle. Ki-67 protein expression was undetectable during the first 18 h after the cells were restimulated to enter the cell cycle and reached the maximal expression around 30 h, with the majority of cells in G2 phase or mitosis^26^. Furthermore, D-type cyclins D1, D2, and D3 (cyclin D) regulate the cell cycle, metabolism, fat cell differentiation, and migration^27,28^. In highly proliferating cells, both D cyclins and Ki67 are present at high levels, unlike in quiescent cells^29^. Our findings demonstrated enhanced gene expression of D-type cyclins D1 (*CCND1*) in ADSCs treated with SAHA, which aligns with another study that quantified the mRNA and protein levels of *CCND1* in placenta derived mesenchymal stem cells with high proliferation capacity^30^. In addition, immortalized canine ADSCs transduced to overexpress *CCND1* and cyclin-dependent kinase 4 (*CDK4R24C*) showed a dramatic increase in proliferation without cellular senescence compared to the primary ADSCs^31^.

Histone modifications have been linked to gene expression, the cell cycle, DNA replication, damage, and repair processe. Some HDACi induce differentiation while others promote self-renewal^32^. In our cell model, ASC52 were treated with SAHA at 40–50% confluency to ensure they were actively dividing, thus avoiding any contact inhibition that might interfere with the drug potency. In this context, SAHA-treated cells showed an increase in the G2 phase of the cell cycle, with no significant changes observed in the sub-G1 population. This suggests that, at least in ADSCs, SAHA primarily induces growth arrest without notable apoptosis. Similar results were observed in human prostate cancer cell lines DU145 and PC-3, where SAHA treatment caused dose-dependent G2/M cell cycle arrest. However, in these cells, apoptosis was noted, potentially indicating differences between transformed cancer cells and non-transformed cells, such as ADSCs^33^. In another study, MI192, a selective inhibitor of HDAC2 and HDAC3, accelerated the osteogenic differentiation of hDPSCs, leading to enhanced histone acetylation and cell cycle arrest at the G2/M phase^34^. Thus, it appears that HDACi induce growth arrest in the G2 phase; however, the outcome, whether apoptosis or differentiation is cell type dependent. This highlights the importance of understanding the cellular context when evaluating the effects of HDACi for therapeutic applications.

ADSCs are known for their capacity for DNA repair, with several markers associated with DNA repair processes. The p53/p21(CDKN1A) pathway potentially allows cells to tolerate external triggers from treatment and activates the repair system to avoid apoptosis, enabling progression through the G1 phase and entry into the S phase of the cell cycle^35,36^. Additionally, while histone modifications can activate DNA damage, DNA damage itself triggers cell cycle checkpoints, which are crucial for slowing down cell cycle progression and facilitating repair^37^. DNA repair is a complex process involving multiple proteins. PCNA is a key factor that transiently accumulates at sites of DNA damage and initiates recombination-associated DNA synthesis after injury. An increase in PCNA levels may be induced by growth factors or as a result of DNA damage in non-cycling cells^38^. To gain further insight into the regulation of SAHA on cell proliferation, we investigated PCNA protein expression. Our results revealed a significant reduction in PCNA levels in ADSCs treated with SAHA, suggesting that these cells may require more time to proliferate in order to recover from DNA damage. Similar findings regarding another HDACi, VPA, were reported by Najafipour et al., indicating that the proliferation capacity of ADSCs was also affected by the treatment. While studying their potential to differentiate into cardiomyocytes, the cells gradually proliferated and reached 70–80% confluency over four weeks^39^. Another study has reported that goat ADSCs treated with 500 nM TSA and 16 μM SAHA showed an increase in H3K9 acetylation and decreased PCNA expression, which inhibits DNA replication and results in G0 & G1 cell cycle arrest^40^.

Several in vivo and in vitro studies have revealed that human mesenchymal stem cells are particularly resistant to apoptosis caused by DNA damage^41^. Furthermore, hematopoietic stem cells and mammary stem cells exhibit a unique, CDKN1A-dependent DNA damage response that inhibits *p53* activation and prevents cell growth arrest^42^. *CDKN1A* halts the progression of the cell cycle at the G2/M checkpoint, while *MDM2,* another well-known target gene of p53, delays the cell cycle progression through the G2/M phase is and inhibiting MDM2 protein expression synergistically increase p53 activation in response to DNA damage. Many drugs have been discovered to reactivate the p53 gene by limiting MDM2 association with p53, inhibiting MDM2 can reactivate p53 and is a potential cancer therapeutic method^43,44^. Our microarray gene expression results showed a significant upregulation of *CDKN1A* and *MDM2*, accompanied by a slight decrease in *p53* expression in response to SAHA treatment. Consistent with these findings, our Western blotting analysis demonstrated increased of p53, p21, accompanied by a decrease in MDM2 protein level, suggesting that SAHA stabilizes p53 levels, leading to the activation of p21 and MDM2 gene expression. It appears that p53 has an antiproliferative function in differentiated cells, as it regulates the transcription of proliferative genes in embryonic stem cells. This may explain why high levels of p53 are associated with the efficient multiplication of stem cells^45^. Additionally, p53 protein expression was reported to play a role in cadiogenic gene activation in HDAC1 depleted cardiac mesenchymal stromal cells (CMCs), establishing a direct biological relationship between p53 signaling and lineage transcriptional activation^46^. In another study *P53-*deficient cells were shown to differentiate to endoderm with high efficiency, indicating an unexpected role in regulating precursor or stem cell differentiation programs. P53 appears to enforce a “differentiation checkpoint” during early endoderm differentiation, which alters cell fate^45^. Moreover, the cellular process derived from our microarray data, as identified by gene ontology analysis, provided evidence for the activation of development process and repression of gene expression related to the cell cycle G2/M phase transition. Enhancing stem cell differentiation capacity is a challenging endeavor; however, it can be effectively improved through histone modification^47^.

Interestingly, the proteomics profile of ADSCs treated with SAHA showed downregulation of pathways related to the cell cycle, DNA replication and ATP-dependent chromatin modeling. The latter involves complexes that disrupt or alter histone-DNA interactions, helping in the establishment of tissue identity, especially at the regulatory sites of specific transcription factors^48^. ATP-dependent chromatin remodeling is tightly regulated in stem cells to balance pluripotency maintenance and differentiation through regulation of chromatin structure and gene expression^49^. In the context of cell differentiation, ATP-dependent chromatin remodeling, and DNA replication are considered important mechanisms that regulate gene expression and establish cellular identity^50^. To this end Marcotte et al. provided supportive evidence of SAHA effects on cutaneous wound healing through affecting endogenous adipose derived stem cells to promote the regeneration process^51^.

Although ADSCs showed great potential for future clinical use in regenerative medicine, they exhibit certain limitations, including restricted in vitro growth and a tendency to replicate aging patterns. Using SAHA as pharmacoepigenetic agent, at nanomolar concentrations could alter ADSCs fate determination through various biological processes in a coordinated manner. This approach could allow for the effective development of functional and safe direction for regenerative therapies. Nevertheless, in vivo studies are essential to validate our findings and to further explore the epigenetic mechanisms of resident stem cells in various tissues, including the integumentary system, which can support wound healing process.

## Materials and Methods

### Cell culture and histone deacetylase inhibitor treatment regimen

**Fig. 6:**
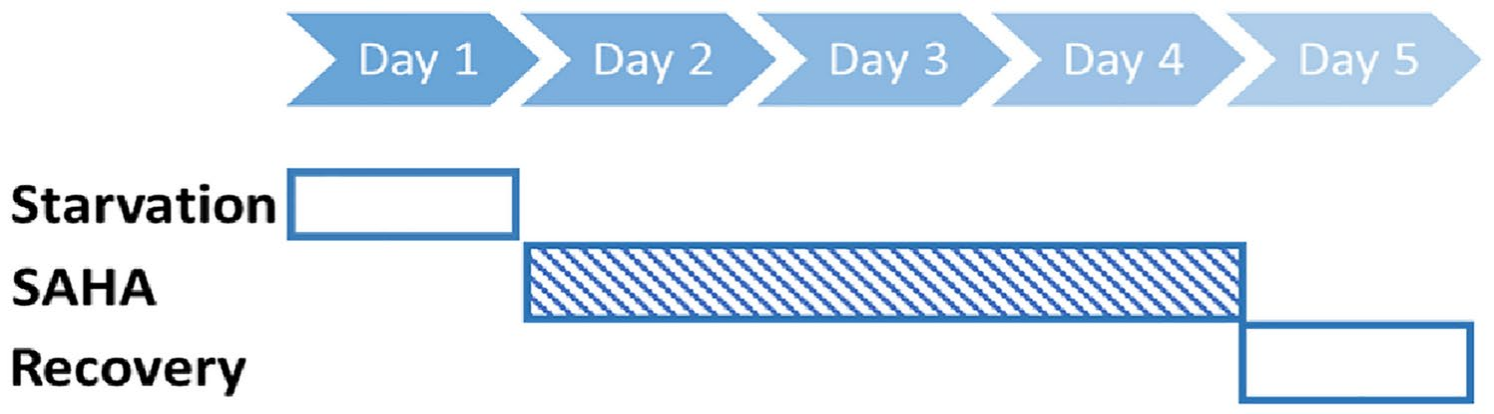
A diagram of ADSCs treatment regimen with SAHA.

ASC52telo cells (ASC52), adipose-derived mesenchymal stem cells immortalized with hTERT (SCRC-4000) were purchased from ATCC, USA and was maintained in a humidified chamber set at 37 °C with 5% CO_2_, using Dulbecco’s Modified Eagle’s Medium (DMEM); (Sigma-Aldrich, Germany) enriched with 10% fetal bovine serum albumin (FBS); (Sigma-Aldrich, Germany). ADSCs were initially seeded at 50% confluency, subjected to 24 h of serum deprivation, followed by treatment with SAHA (Abcam, UK) at concentrations of 10, 100, 1000, and 2000 nM. SAHA was dissolved in EtOH, and the latter was considered as a control vehicle. The treatment was applied sequentially for 72 h. Following the treatment, fresh DMEM media was added for 24 h. The treatment regimen is shown in Fig. 6.

### Cell viability assessment

Cells were seeded in 96-well plates at a density of 5000 cells per 200 µl per well. Cell viability was assessed after treating the cells with the previously mentioned concentrations or EtOH, while cells that received no treatment served as a control. 5 mg/ml MTT salt (3-(4,5-dimethylthiazol-2-yl)-2,5-diphenyltetrazolium bromide); (Sigma-Aldrich, Germany) were added to the cells for 3 h in CO_2_ incubator. MTT reduction reaction was stopped by using 100 µl Dimethyl sulfoxide (DMSO); (Sigma-Aldrich, Germany) as a solubilization solution. Absorbance readings were taken at 570 nm using a Molecular Devices SpectraMax Plus 384 microplate reader (VWR, USA).

### Cell cycle progression analysis

Cells were initially seeded at 50% confluency and then subjected to treatment with either 1 µM SAHA or EtOH as a control vehicle. Additionally, cells that received no treatment served as a control group. On the day of cell harvesting, media containing dead cells was collected, and the cells were detached using trypsin and added to collected media. The cell suspension was then centrifuged at 1200* g* for 5 min and subsequently fixed by incubating in 70% pre-chilled ethanol, followed by storage at − 20 °C. Fixed cells were washed with PBS, pelleted, and resuspended in a PI solution containing (50 µg/mL) and (100 µg/mL) of RNase A and incubated for 30 min at 37 °C. The PI-stained cells were analyzed using Beckman Coulter Gallios Flow Cytometer (Beckman, USA). Blue laser with an excitation wavelength of 488 nm was employed, and PI fluorescence was detected using the red channel (560-615 nm). Low flow rate was used, and approximately 1 × 10^5^ nuclei were examined in each sample. Qualitative gating was applied to eliminate clumps and aggregates, using a dot blot with PI fluorescence area (PI-A) vs. PI fluorescence width (PI-W) displayed on a linear scale. The acquisition settings remained consistent throughout the experiment. A PI-A histogram was utilized to visualize the nuclear DNA content. Data analysis was performed using ModFit LT™ software (Verity Software House, USA).

### SDS–polyacrylamide gel electrophoresis and immunoblotting

Whole-cell lysates were isolated as previously reported with brief modifications^52^. Radioimmunoprecipitation assay buffer (RIPA) was used accompanied with 1 × complete protease inhibitor (Roche, Switzerland). Total protein was quantified using Bio-Rad Protein Assay (Bio-Rad Laboratories, USA), and read at 750 nm in SpectraMax Plus 384 Microplate Reader (Molecular Devices, USA). Twenty-five μg of protein was loaded onto 4–12% Bis–Tris precast polyacrylamide gels (Bio-Rad Laboratories, USA) and subjected to electrophoresis for 90 min at 150 V in the Bio-Rad PowerPac™ HC system (Bio-Rad, USA). Subsequently, they were transferred to nitrocellulose membranes using the Trans-Blot Transfer Turbo System (Bio-Rad Laboratories, USA). Afterwards, the membranes were blocked in a 5% skimmed milk solution for 1 h at room temperature and probed with primary antibodies for Histone deacetylase (HDAC) antibody sampler kit (cell signaling technology, USA), including HDAC1, HDAC2, HDAC3, HDAC4, HDAC6, and PCNA, P21 Waf1/Cip1, MDM2(SMP14), p53(DO-1), p27 Kip1 (D37H1), and β-Actin overnight at 4 °C, following the manufacturer’s recommendations. The membranes were washed three times with 0.1% Tween-20 for 10 min each, then incubated with corresponding secondary antibodies (1:1000) in the darkness for 1 h. Following washing, chemiluminescent signal was detected using ECL Western Blotting Detection Reagents (Bio-Rad Laboratories, USA) and scanned in ChemiDoc imaging system (Bio-Rad Laboratories, USA). Densitometric analysis was conducted using Image Lab, version 6.0.1 (Bio-Rad Laboratories, USA).

### Immunofluorescence staining and imaging

Cells were fixed and permeabilized using 4% paraformaldehyde (Histolab Products AB, Sweden) for 15 min and 0.5% TritonX-100 (Sigma-Aldrich, Germany) for 10 min respectively. The cells were incubated with a blocking solution containing 3% BSA for 1 h, followed by overnight incubation with monoclonal rabbit anti-human Ki-67/MKI67 (Novus Biologicals Inc, USA); (1:250) at 4 °C, followed by 1 h incubation in the darkness with the secondary polyclonal goat anti-rabbit labeled with Alexa Fluor® 488 (Abcam, USA); (1:1000). The cells were counterstained with NucBlue™ Fixed Cell Stain (Life Technologies Corporation, USA) to visualize the nucleus, and mounted onto microscope slides using ProLong™ Glass Antifade Mountant (Thermo Fisher Scientific, USA) according to the manufacturers recommendations. The slides were then examined under a confocal microscope (Carl Zeiss LSM 700, Germany) with a Plan-Apochromat 63x/1.4 oil immersion objective lens. Images were captured using the ZEN 2011 SP3 software and analyzed with imageJ software (NIH, USA).

### Microarray gene expression analysis

Total cellular RNAs were extracted using RNeasy Kit (Qiagen, Germany), following the manufacturer’s instructions. RNA concentration quantified and A260/A280 ratio were determined using the NanoDropTM 1000 spectrophotometer (Thermo Fisher Scientific, USA). RNA quality was checked using Agilent Bioanalyzer 2100 RNA (Agilent, USA)^28^. The samples with RNA integrity number (RIN) equal to 9 were hybridized with Human Clariom™ S array (Thermo Fisher Scientific, USA) following the manufacturer’s protocols. The arrays were washed, stained using GeneChip Fluidics Station 450 (Thermo Fisher Scientific, USA), and scanned with GeneChip Scanner 3000 7G (Affymetrix, Santa Clara, CA, USA).

Arrays were processed with the oligo package to summarize probe-set transcript clusters. One sample from EtOH group was removed because the intensity was low (mean = 36.49, sd = 56.19). Raw RNA expression data were normalized using the robust multi-array average (RMA) method applied through the oligo package^53^. Hierarchical clustering was carried out, while heatmaps of significantly regulated genes were plotted using the pheatmap v1.0.12 package^54^. Annotation of probes was performed using clariomshumantranscriptcluster.db package. The Limma R package^56^ was used to identify the differentially expressed genes (DEGs), adjusting the associated *p* value with Benjamini–Hochberg (BH) method to control the false discovery rate. Following the exclusion of one EtOH sample, the control groups (EtOH and untreated) were combined, and the gene expression was compared to the study group (SAHA). The log2 fold change values of probe sets mapped on the same gene were averaged and genes with fold change value |FC |≥ 1.5 and an adjusted FDR *p* value < 0.05 were identified as differentially expressed.

### Reverse transcription and quantitative real-time polymerase reaction (qPCR)

Reverse transcription of 1 µg of total RNA was done using the QuantiTect® Reverse Transcription Kit (Qiagen, Germany) following the manufacturer’s instructions. The gene expression levels were determined using the PowerUp™ SYBR™ Green Master Mix (Thermo Scientific, USA). The primer sequences for the specific genes of interest are detailed in (Table 1). Real-time quantitative PCR was performed using the Applied Biosystems™ 7500 Real-Time PCR System (Applied Biosystem, USA), The reaction temperature profile was as follows: 95 °C for 20 min, 40 cycles of 95 °C for 10 s and 60 °C for 1 min. To calculate the relative fold change in mRNA expression, we utilized the delta delta Ct (ΔΔCt) method, and the results were normalized to GAPDH.

**Table 1:** Primers sequences used for the qualitative polymerase chain reaction (q-PCR).

Accession number	Gene name	Sequence (5'—> 3')	Product length (bp)
NM_053056	Cyclin D1 (CCND1)	F 5′-GAGGAGCTGCTGCAAATGG-3′	58
		R 5′-CGGCCAGGTTCCACTTGA-3′	
NM_001374511.1	Cyclin-dependent kinase inhibitor 1A (p21, Cip1) (CDKN1A/P21)	F 5′-ATGAAATTCACCCCCTTTCC-3′	174
		R 5′-CCCTAGGCTGTGCTCACTTC-3′	
NM_001367990.1	MDM2 proto-oncogene, E3 ubiquitin protein ligase (MDM2)	F 5′-CAGTAGCAGTGAATCTACAGGGA-3′	85
		R 5′-CTGATCCAACCAATCACCTGAAT-3′	
NM_001276696.3	Homo sapiens tumor protein p53 (TP53)	F 5′-ACCTATGGAAACTACTTCCTGAAA-3′	257
		R 5′-GCTGCCCTGGTAGGTTTTCT-3′	

### Mass spectrometry samples preparation

Whole-cell lysates containing protein amounts ranging from 60 to 260 µg were treated with 200 mM dithiothreitol (Sigma, Germany) at 56 °C for 30 min, alkylated with 400 mM iodoacetamide (Sigma, Germany) in the darkness for 60 min, then precipitated overnight at 4 °C with 1:9 volume 95% EtOH with 50 mM sodium acetate (Sigma, Germany). The precipitated samples were centrifuged at 13,000 rpm for 1 h, the supernatants were removed and dried in SpeedVac, then suspended to 0.1 M ammonium bicarbonate (Sigma, Germany) buffer followed by digestion with Trypsin Gold (Promega, UK) at a ratio of 1:50 (protease/protein) at 37 °C overnight^57^. Samples were diluted in 1 M sodium chloride with 1% formic acid (Sigma, Germany) and cleaned through Nanosep® Centrifugal Devices with Omega™ Membrane 30 K filter (Pall corporation, USA) to remove peptides with glycosaminoglycan (GAG) chains. The peptides concentrations were determined using the pierce quantitative colorimetric peptide assay (Thermo Scientific, USA).

### Instrumentation and data-independent acquisition (DIA) analysis

The samples were further spiked with iRT peptides before analyses with mass spectrometry. Digested peptides were separated with nanoflow reversed-phase chromatography with an Evosep One liquid chromatography (LC) system (Evosep, Denmark). Separation was performed with the 30 SPD method (gradient length 44 min) using a 15 cm × 150 µm Evosep column packed with 1.5 μm ReproSil-Pur C18-AQ particles at 40 °C. A captive source mounted on a timsTOF Pro mass spectrometer from Bruker Daltonics was used with timstof control (4.0) and Hystar (6.0) software. Samples were analyzed in DIA-PASEF mode with a m/z range of 400–1201 and an IM range of 0.6–1.43 1/K0 [V s/cm^2^]. DIA-PASEF windows and collision energy had a base of 0.85 1/K0 [V s/cm^2^] set at 20 eV and 1.30 1/K0 [V s/cm^2^] set at 59 eV.

Direct-DIA was conducted using Spectronaut ™ 17.0 software, subsequent protein search was conducted using spectral library. The human protein fasta files were downloaded from the uniprot database (2022-10-12). Human species were selected for all the identified proteins. The protein identification threshold was set at < 1% for both peptide and protein false discovery rates. Precursor quantitation was performed at MS2 level^58^. We excluded proteins with three or more missing values within each biological group to avoid any potential bias. The total count of identified proteins after this exclusion was 6272 proteins. Prior to the analysis, logFC transformation to all intensity values was applied.

The mass spectrometry proteomics data have been deposited to the ProteomeXchange Consortium via the PRIDE partner repository with the dataset identifier PXD053005.

### Gene ontology and enrichment analyses

To identify differentially expressed genes (DEGs), we employed a multivariate linear regression approach and principal components analysis (PCA) with the Limma R package. Heatmap was plotted by ComplexHeatmap (version 2.18.0) R package.Gene ontology (GO) was applied to annotate genes and proteins, which reflects biological processes (BP) and molecular function (MF) for microarray and mass spectrometry data. Gene Set Enrichment was analyzed with ClusterProfiler to evaluate overrepresentation of certain pathways and reactome database was used^59–61^.

### Statistical analysis

All experiments were conducted at a minimum of three independent biological replicates. Data were presented as means with the corresponding standard error of the mean (SEM). Statistical analysis was carried out using GraphPad Prism software (version 9.4.0, USA) employing an unpaired t-test. *p* value < 0.05 was indicating of a statistically significant difference, which presented as follows: * for *p* < 0.05, ** for *p* < 0.01, and *** for *p* < 0.001.

## Supplementary Information

Supplementary Information 1.

Supplementary Information 2.

Supplementary Information 3.

Supplementary Information 4.

Supplementary Information 5.

## Data Availability

The data reported in this study are accessible in the supplementary material and upon request from the corresponding author. The microarray data has assigned a GEO accession numbers GSE269774. The mass spectrometry data are available via ProteomeXchange with identifier PXD053005.
