# Supplementary material for: Modulation of biological activities in adipose derived stem cells by histone deacetylation
Source: Sci Rep. 2025 Jan 29;15:3629. doi: 10.1038/s41598-024-84652-1 (PMC11779964; doi:10.1038/s41598-024-84652-1)
Supplement: Supplementary file 1 — Supplementary Information 1. [file 41598_2024_84652_MOESM1_ESM.pptx]

## Slide 1
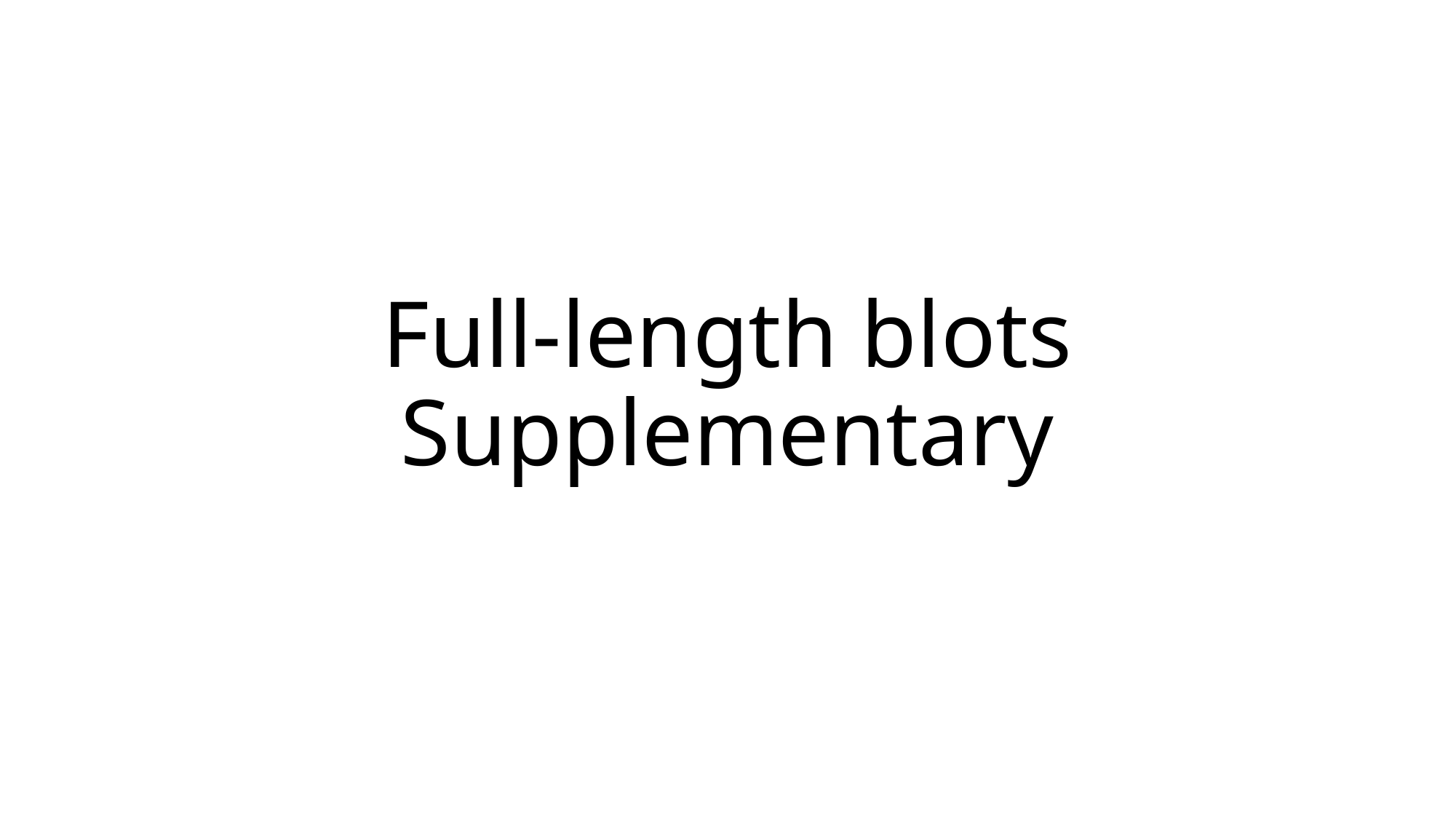

# Full-length blots Supplementary

## Slide 2
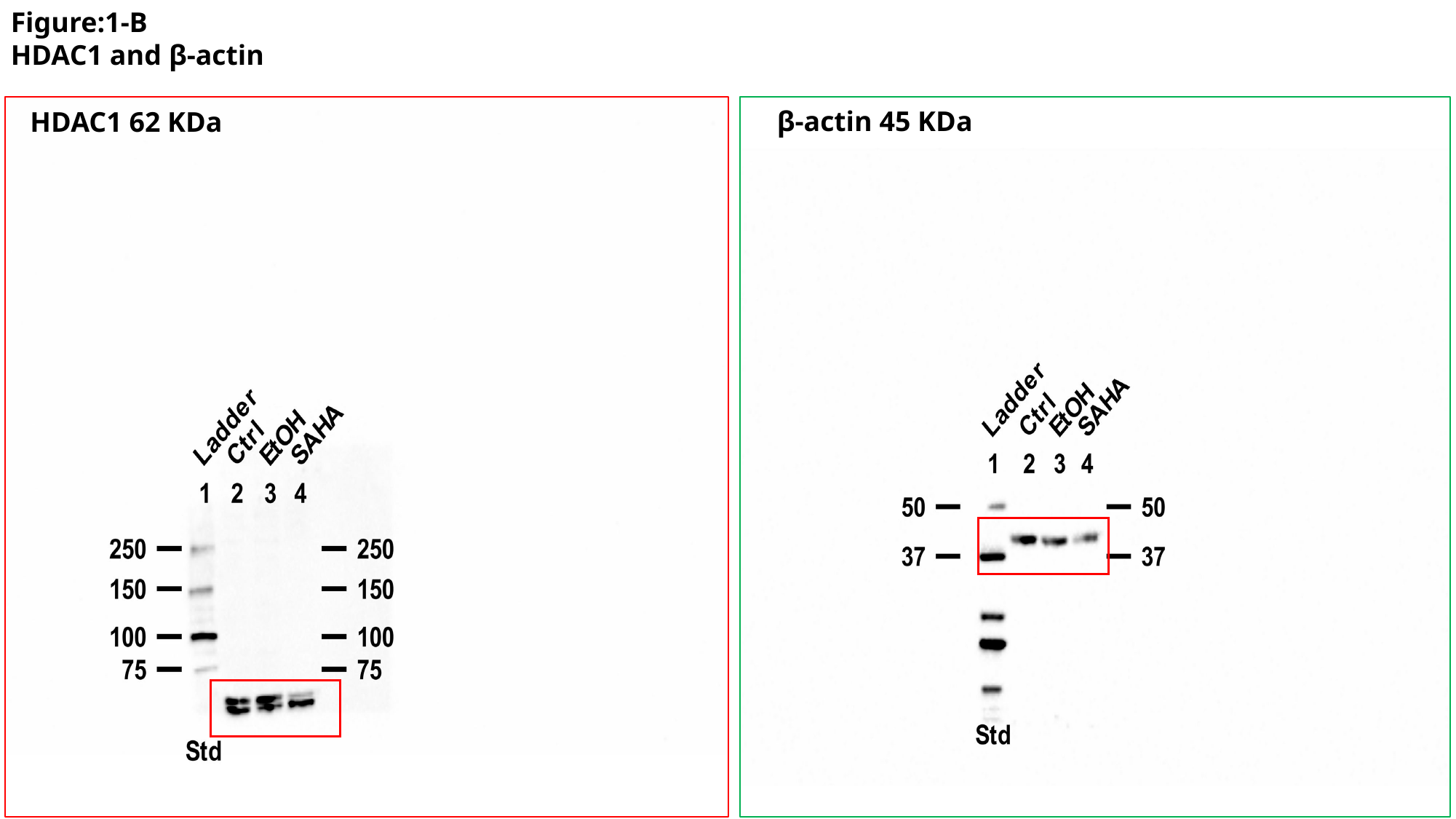

Figure:1-B
HDAC1 and β-actin
HDAC1 62 KDa
β-actin 45 KDa

## Slide 3
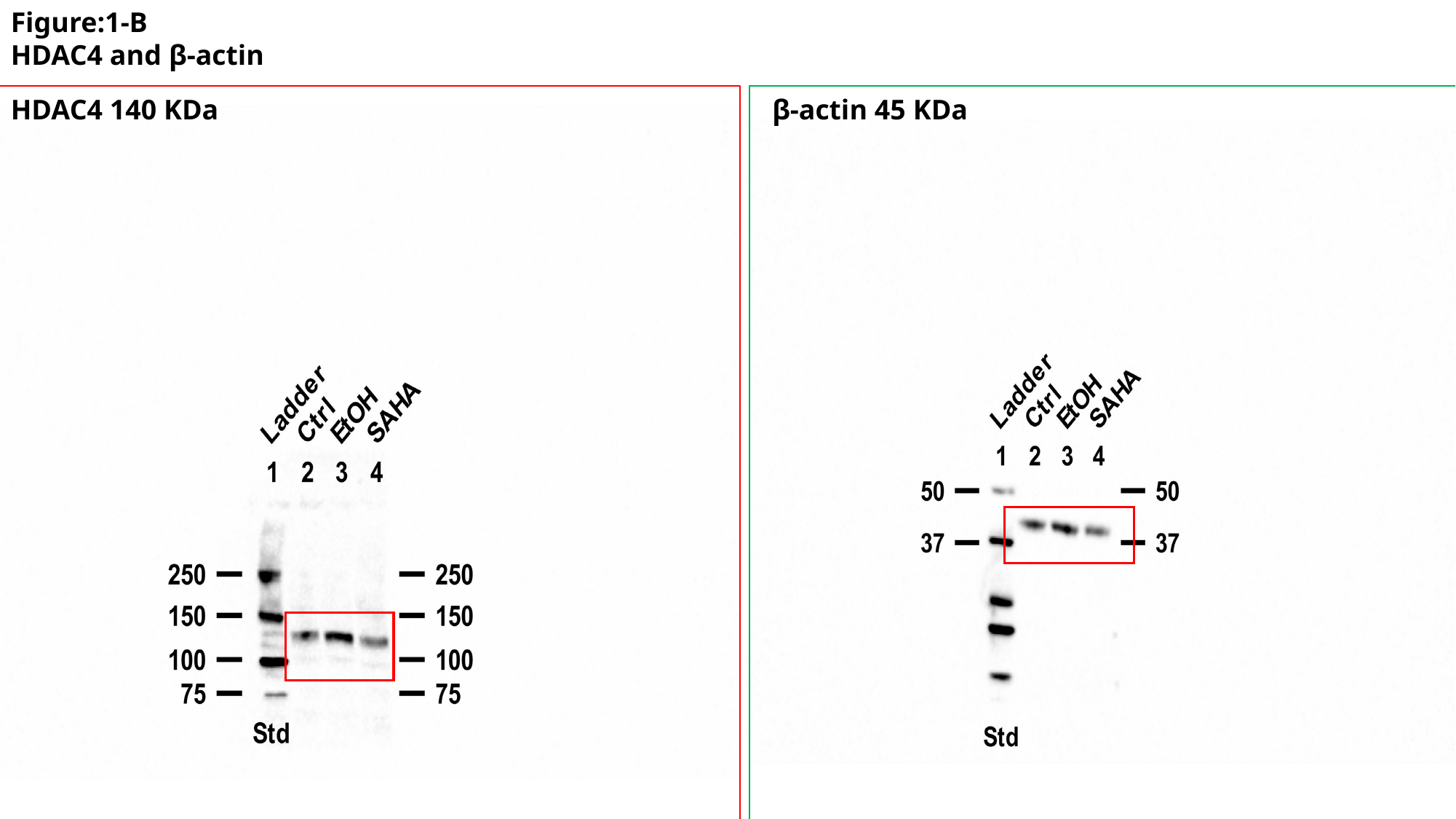

Figure:1-B
HDAC4 and β-actin
HDAC4 140 KDa
β-actin 45 KDa

## Slide 4
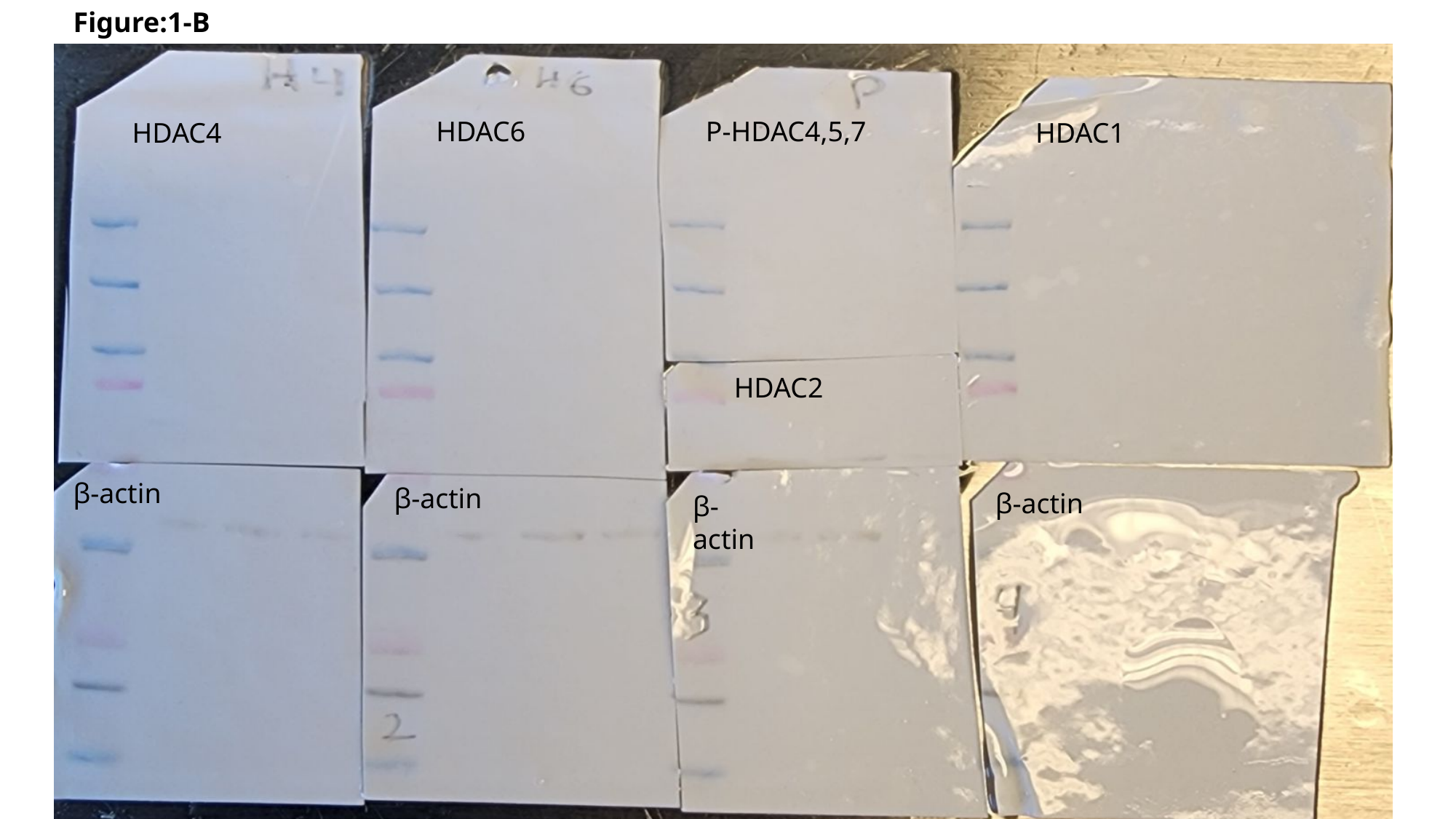

Figure:1-B
HDAC6
P-HDAC4,5,7
HDAC4
HDAC1
HDAC2
β-actin
β-actin
β-actin
β-actin

## Slide 5
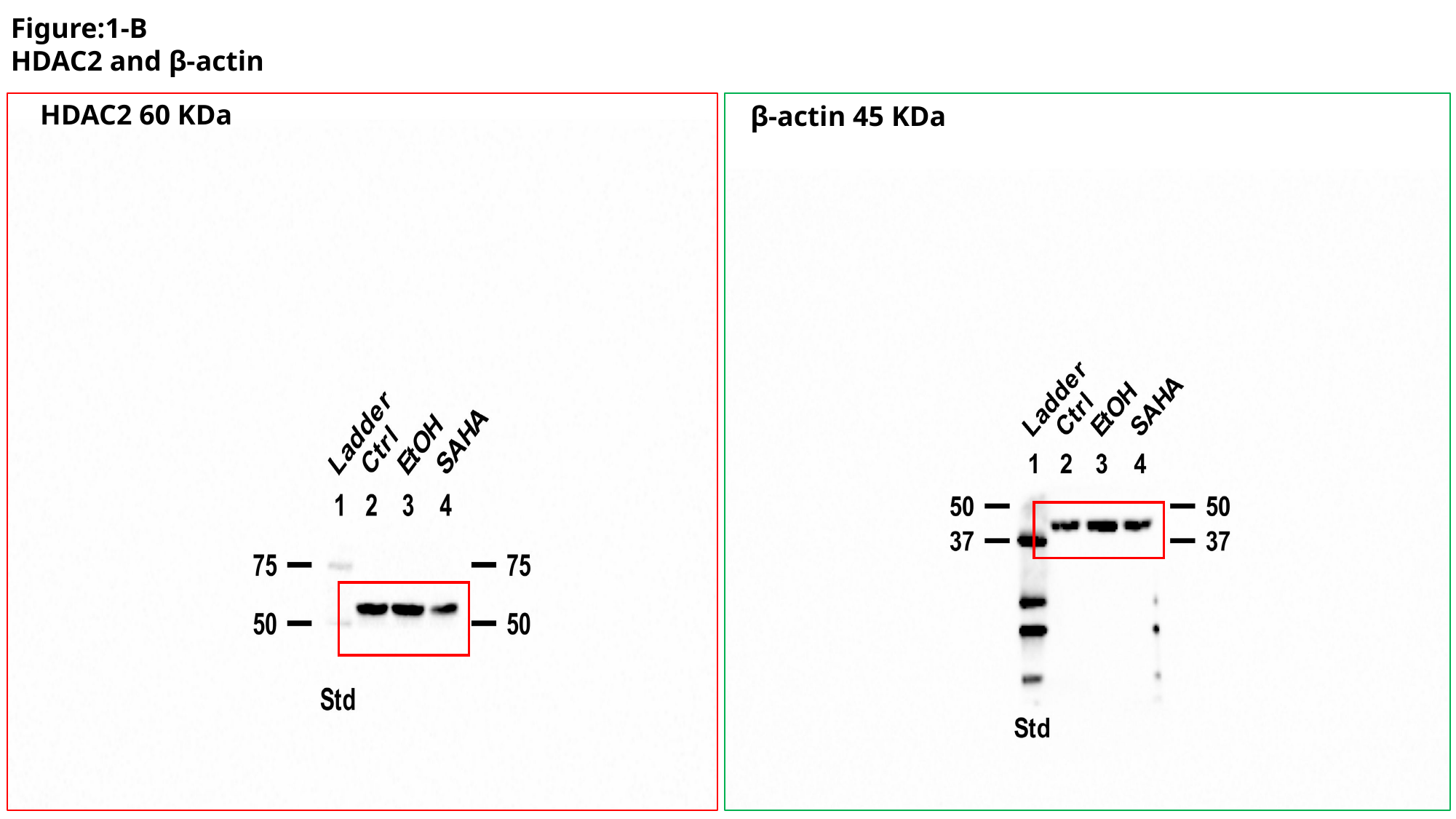

Figure:1-B
HDAC2 and β-actin
HDAC2 60 KDa
β-actin 45 KDa

## Slide 6
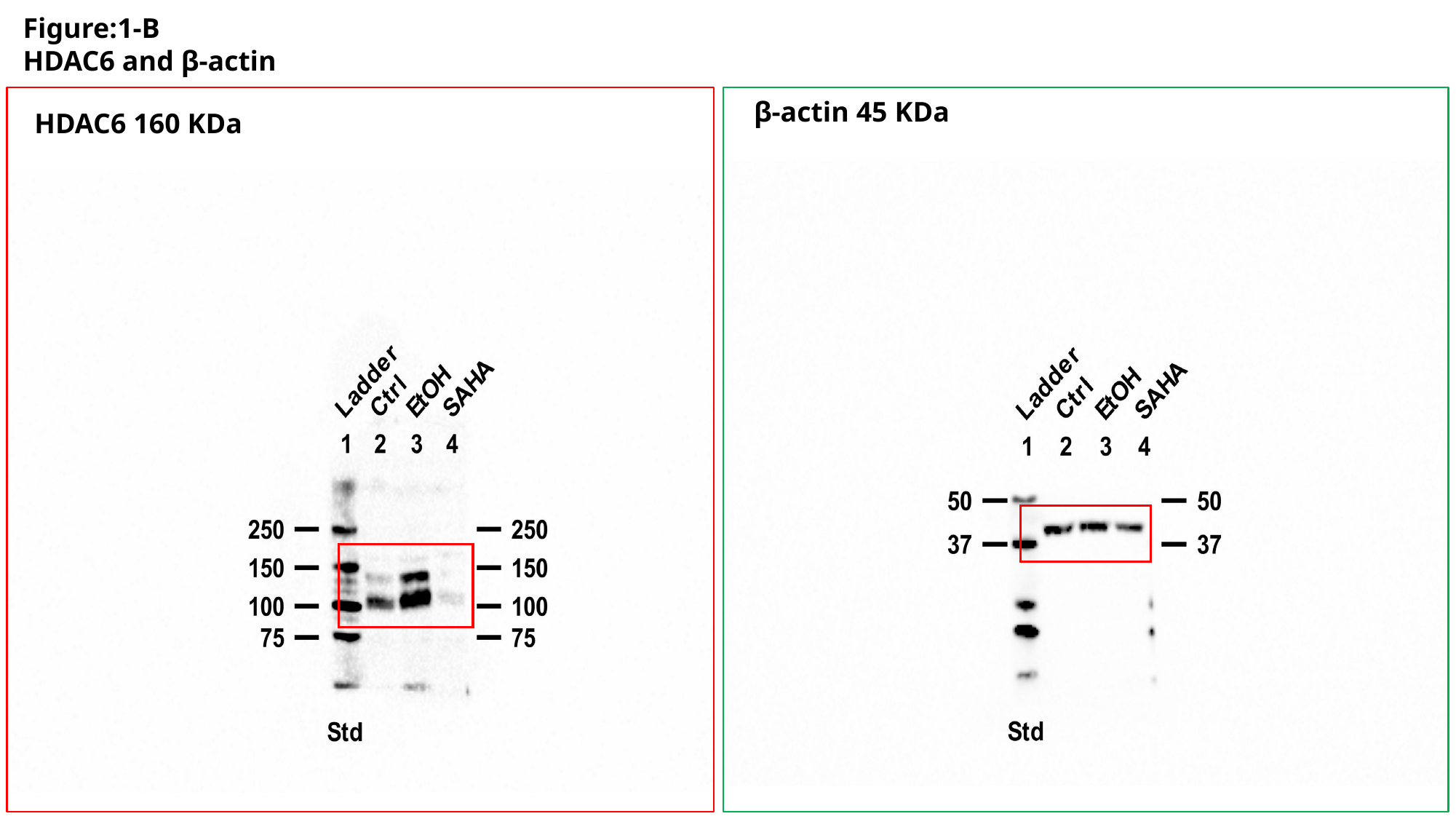

Figure:1-B
HDAC6 and β-actin
HDAC6 160 KDa
β-actin 45 KDa

## Slide 7
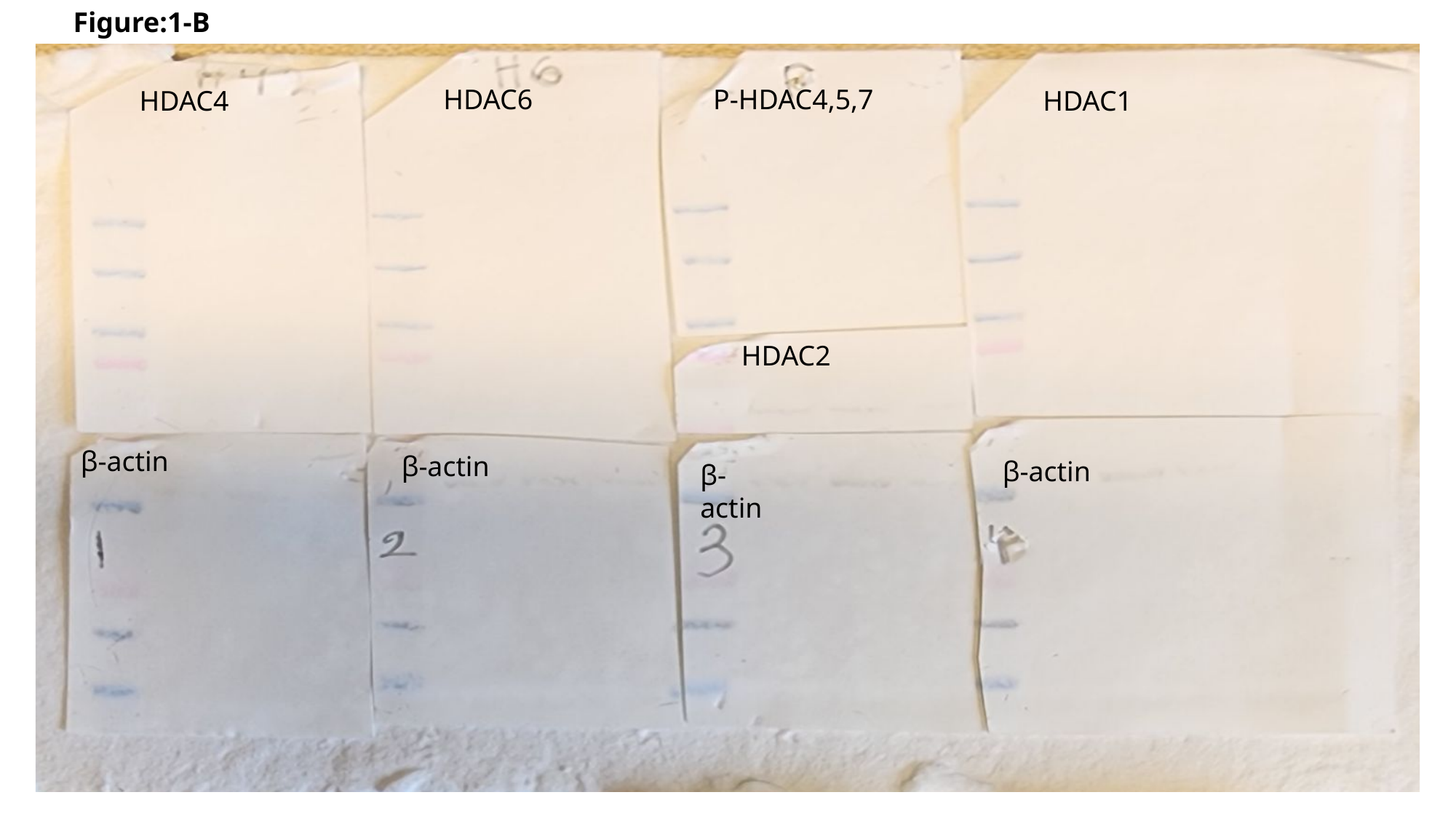

Figure:1-B
HDAC6
P-HDAC4,5,7
HDAC4
HDAC1
HDAC2
β-actin
β-actin
β-actin
β-actin

## Slide 8
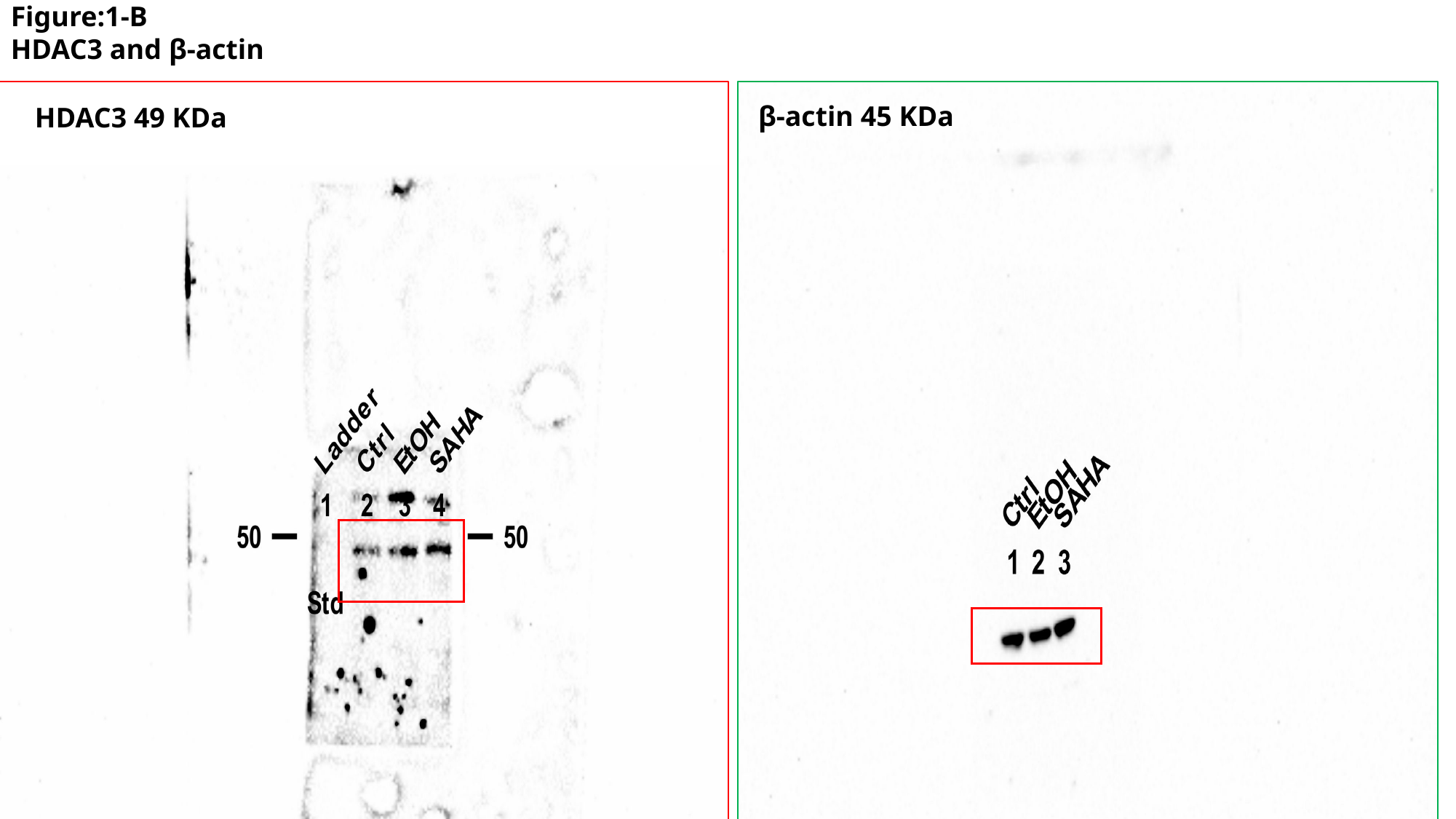

Figure:1-B
HDAC3 and β-actin
HDAC3 49 KDa
β-actin 45 KDa

## Slide 9
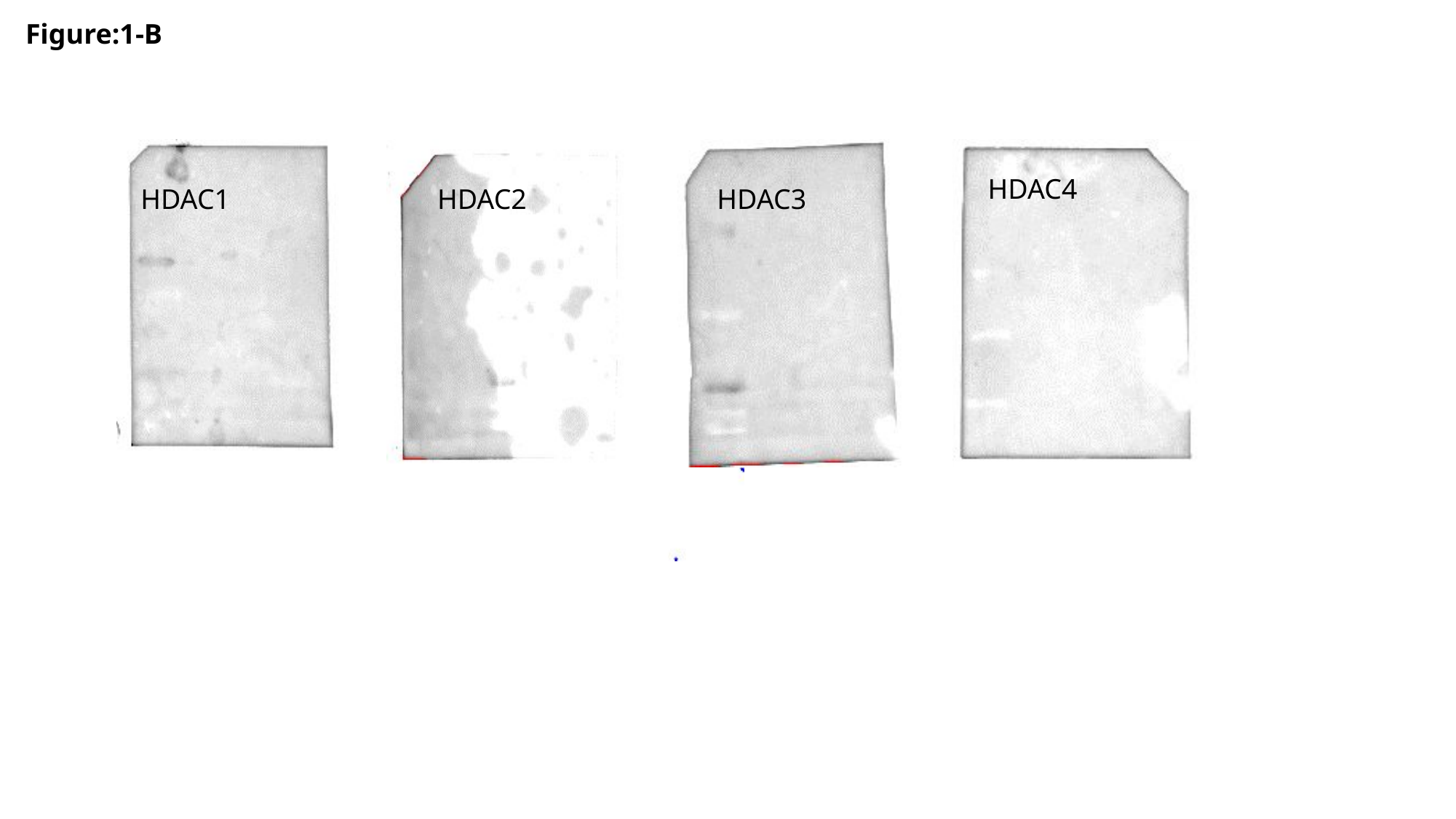

Figure:1-B
HDAC4
HDAC1
HDAC2
HDAC3

## Slide 10
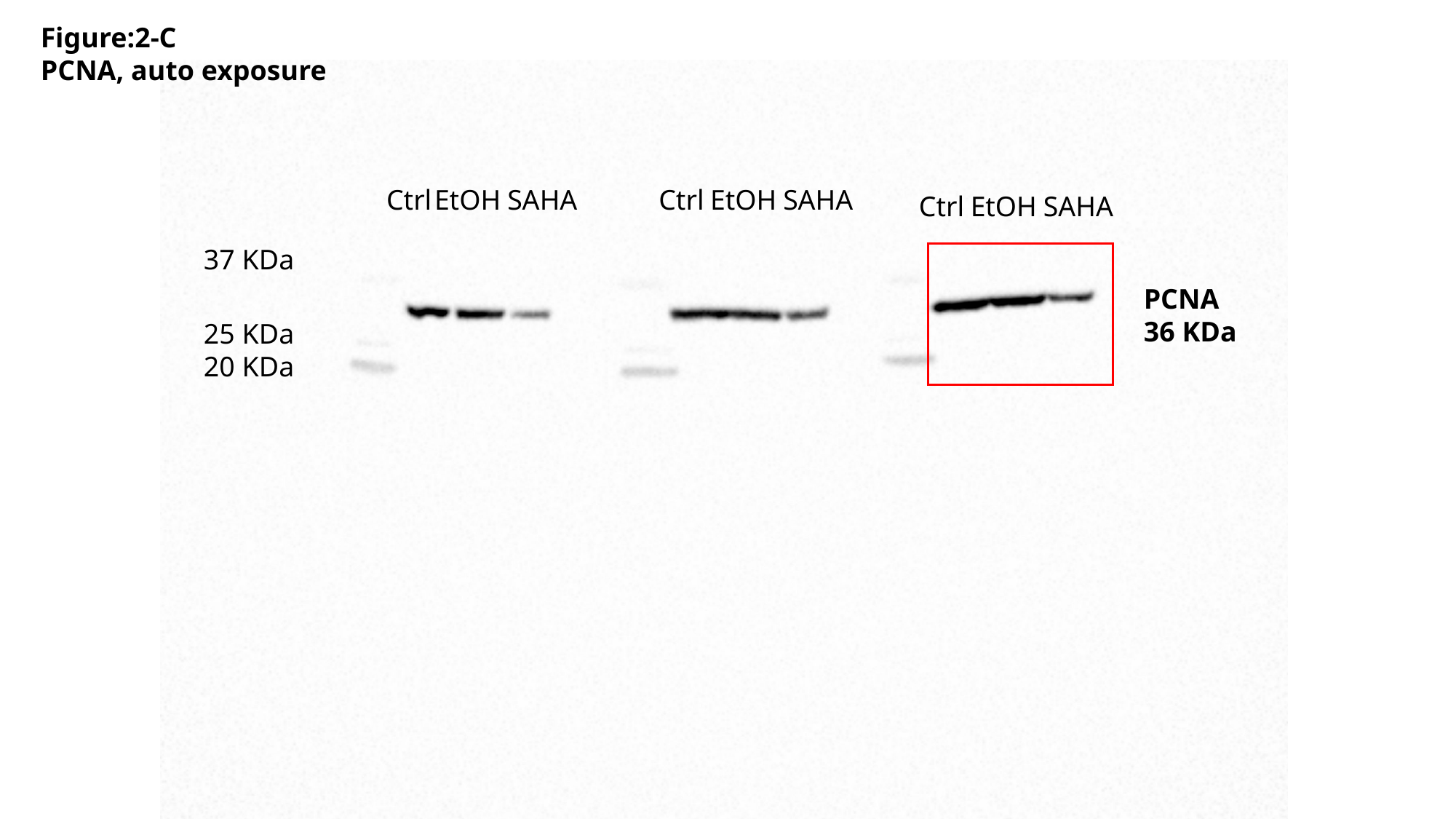

Figure:2-C
PCNA, auto exposure
Ctrl
EtOH
SAHA
Ctrl
EtOH
SAHA
Ctrl
EtOH
SAHA
 37 KDa
PCNA
36 KDa
 25 KDa
 20 KDa

## Slide 11
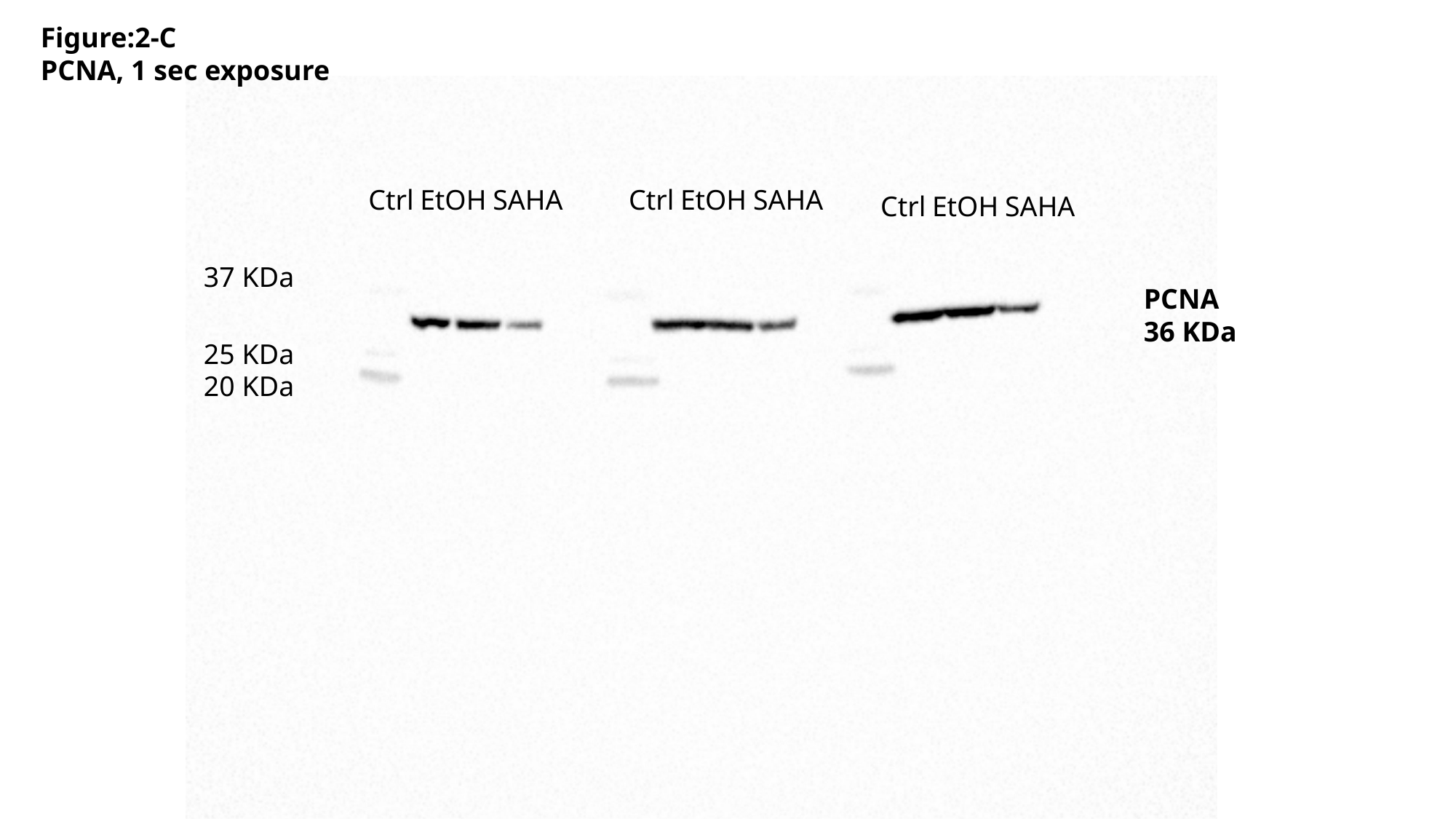

Figure:2-C
PCNA, 1 sec exposure
Ctrl
EtOH
SAHA
Ctrl
EtOH
SAHA
Ctrl
EtOH
SAHA
 37 KDa
PCNA
36 KDa
 25 KDa
 20 KDa

## Slide 12
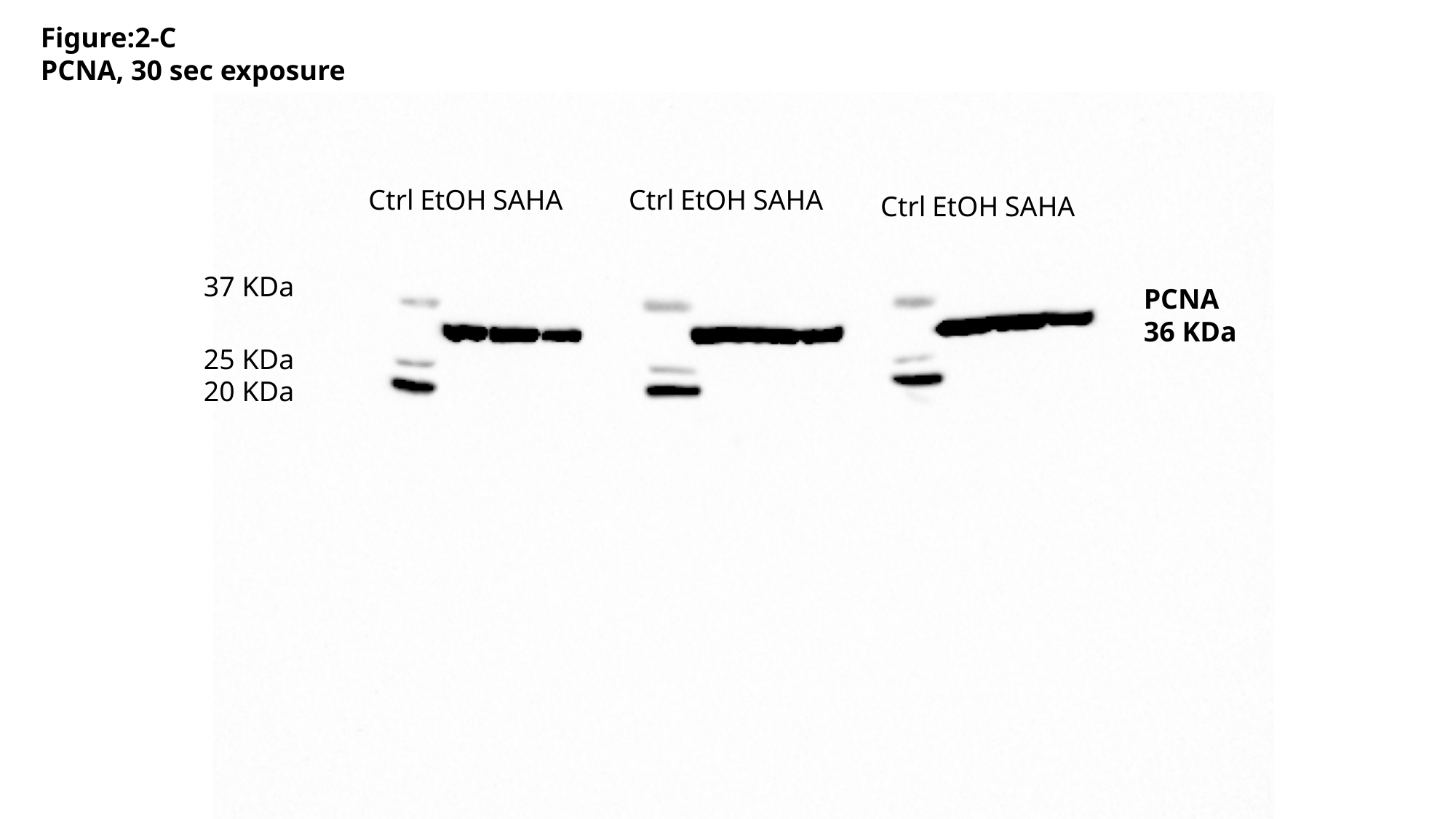

Figure:2-C
PCNA, 30 sec exposure
Ctrl
EtOH
SAHA
Ctrl
EtOH
SAHA
Ctrl
EtOH
SAHA
 37 KDa
PCNA
36 KDa
 25 KDa
 20 KDa

## Slide 13
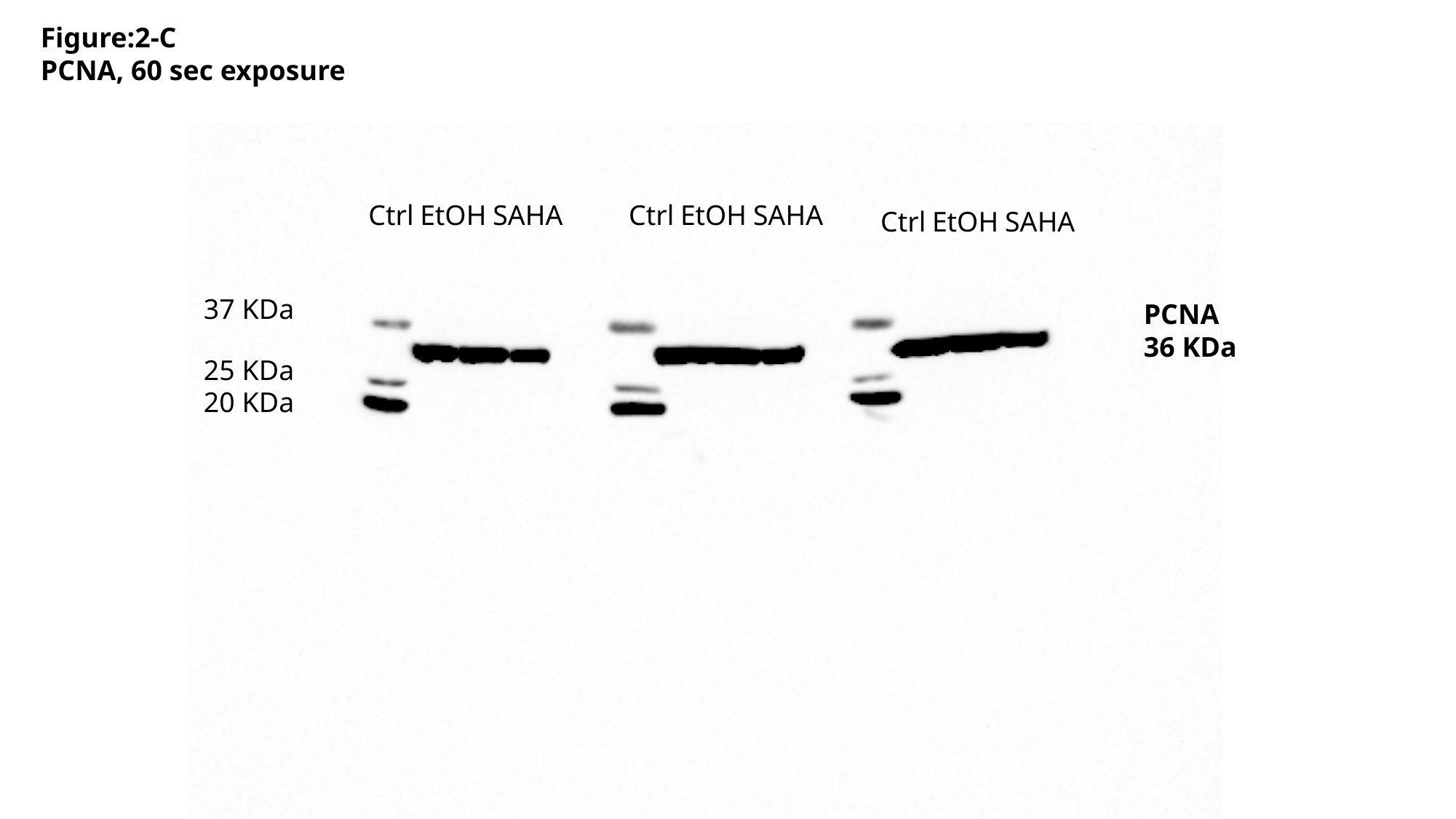

Figure:2-C
PCNA, 60 sec exposure
Ctrl
EtOH
SAHA
Ctrl
EtOH
SAHA
Ctrl
EtOH
SAHA
 37 KDa
PCNA
36 KDa
 25 KDa
 20 KDa

## Slide 14
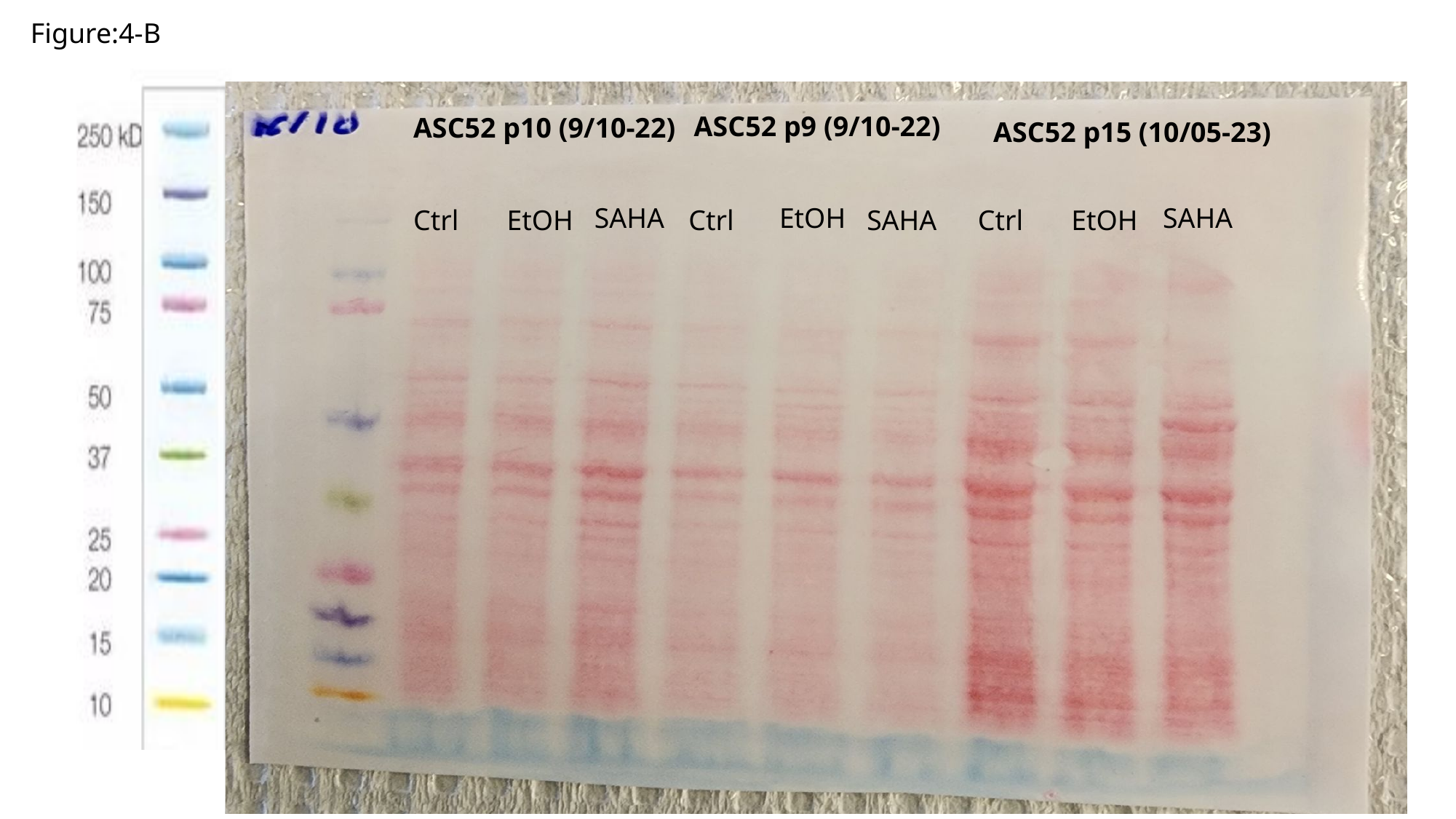

# Figure:4-B
ASC52 p9 (9/10-22)
ASC52 p10 (9/10-22)
ASC52 p15 (10/05-23)
SAHA
EtOH
SAHA
Ctrl
EtOH
Ctrl
Ctrl
EtOH
SAHA

## Slide 15
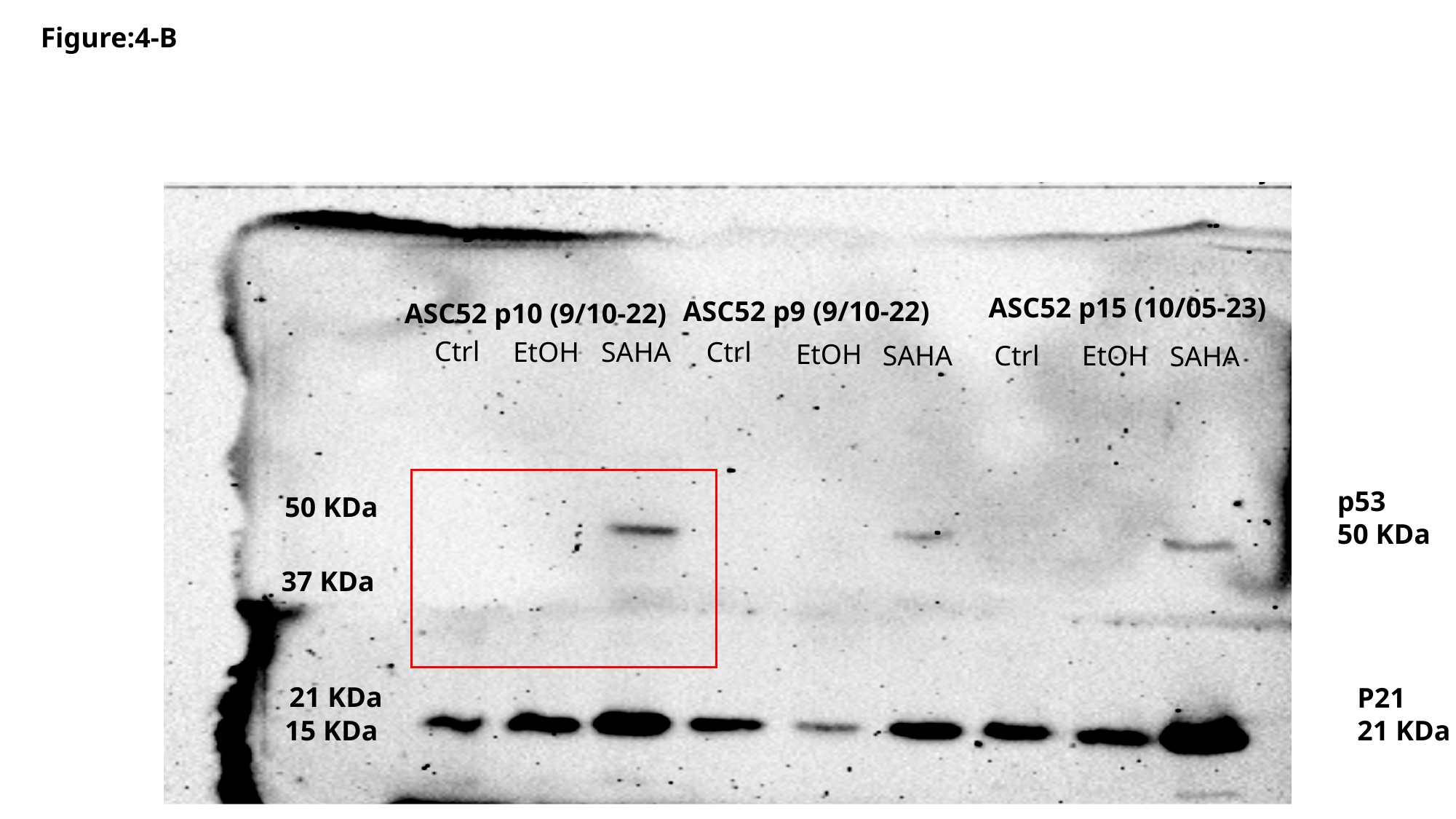

Figure:4-B
ASC52 p15 (10/05-23)
ASC52 p9 (9/10-22)
ASC52 p10 (9/10-22)
50 KDa
37 KDa
21 KDa
15 KDa
Ctrl
EtOH
SAHA
Ctrl
EtOH
SAHA
Ctrl
EtOH
SAHA
p53
50 KDa
P21
21 KDa

## Slide 16
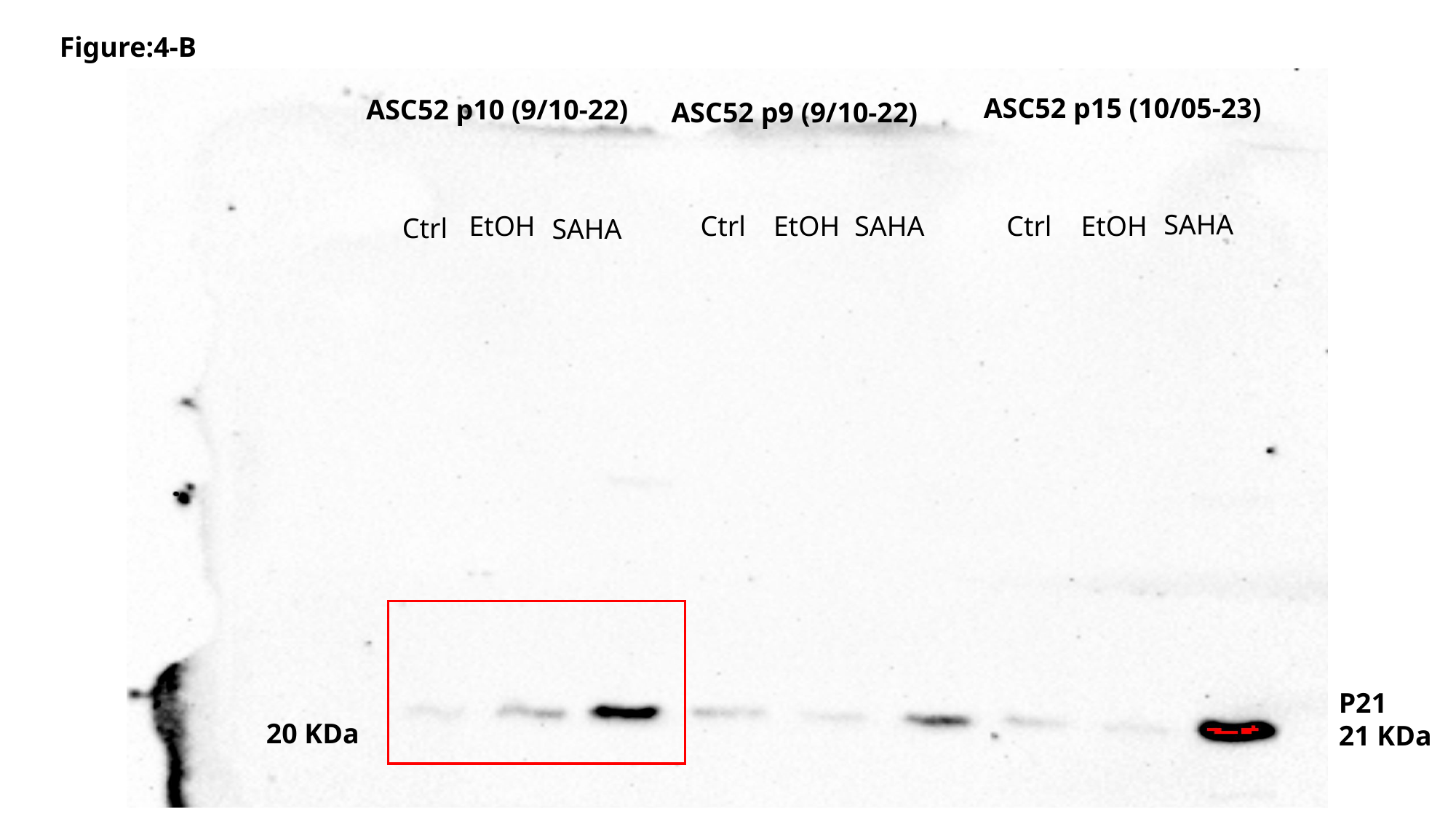

Figure:4-B
ASC52 p15 (10/05-23)
ASC52 p10 (9/10-22)
ASC52 p9 (9/10-22)
SAHA
EtOH
Ctrl
EtOH
SAHA
Ctrl
EtOH
Ctrl
SAHA
P21
21 KDa
20 KDa

## Slide 17
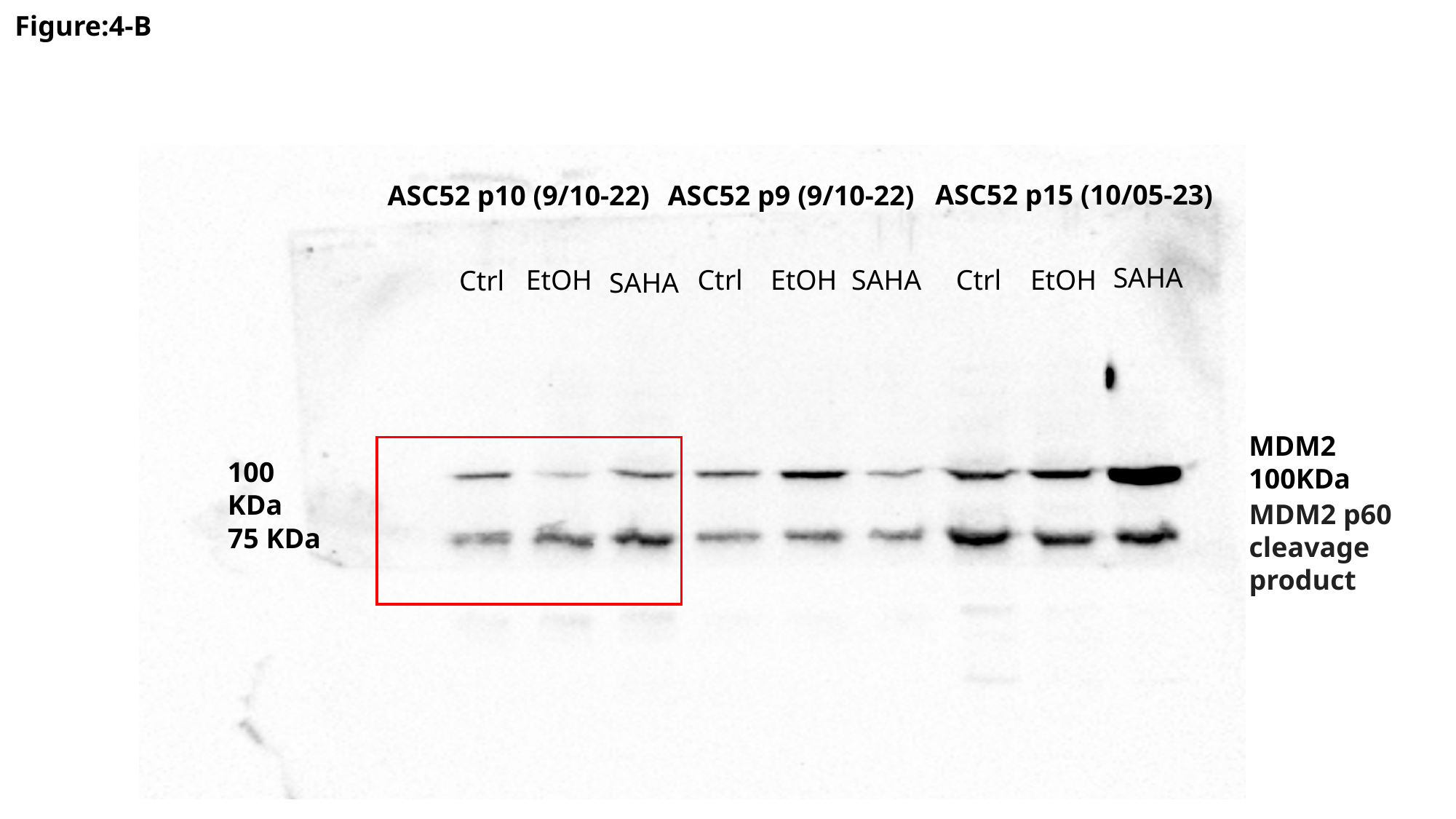

Figure:4-B
ASC52 p15 (10/05-23)
ASC52 p10 (9/10-22)
ASC52 p9 (9/10-22)
SAHA
EtOH
Ctrl
EtOH
SAHA
Ctrl
EtOH
Ctrl
SAHA
MDM2
100KDa
100 KDa
MDM2 p60 cleavage product
75 KDa

## Slide 18
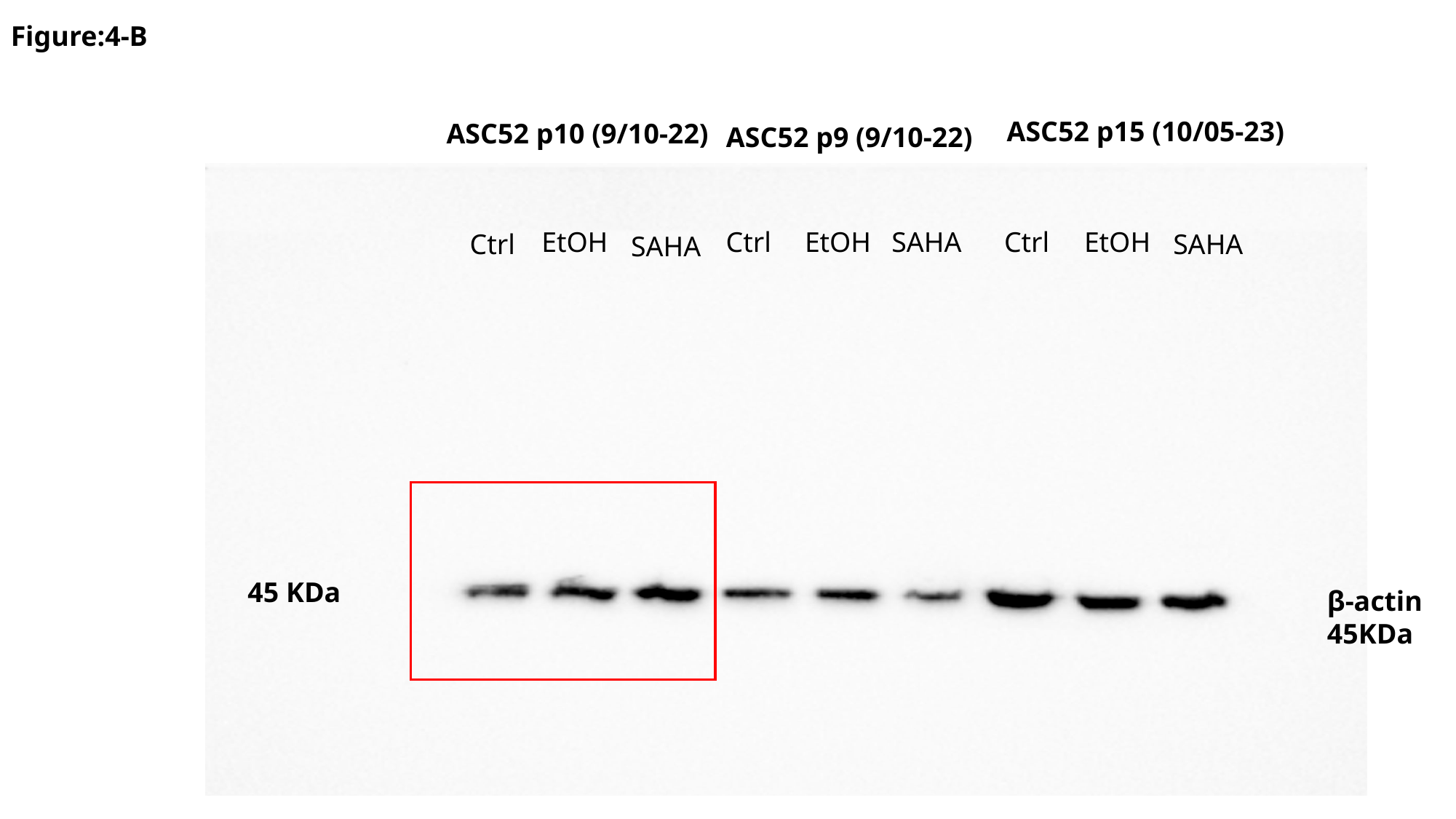

Figure:4-B
ASC52 p15 (10/05-23)
ASC52 p10 (9/10-22)
ASC52 p9 (9/10-22)
EtOH
Ctrl
EtOH
SAHA
Ctrl
EtOH
Ctrl
SAHA
SAHA
45 KDa
β-actin
45KDa

## Slide 19
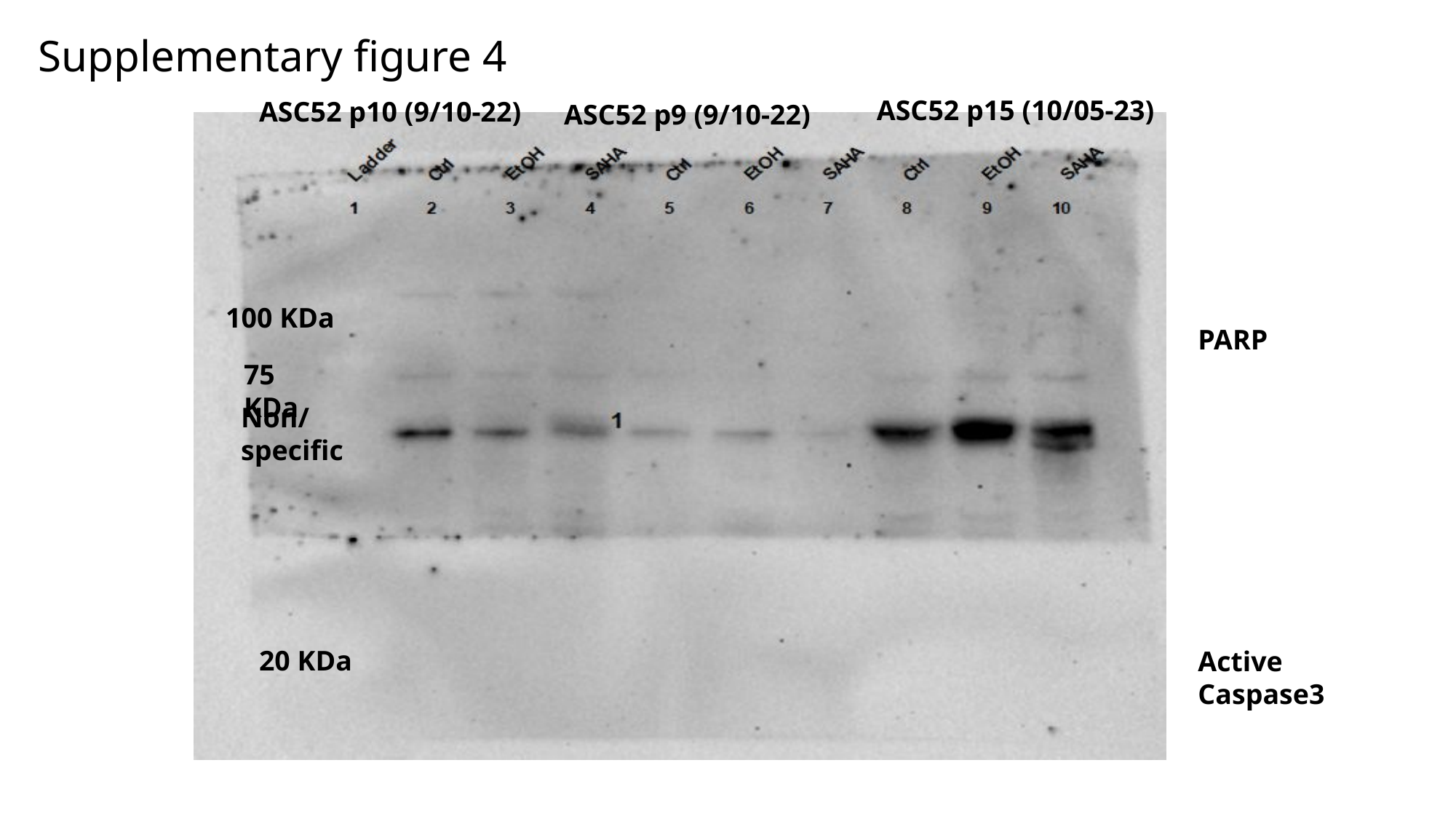

Supplementary figure 4
ASC52 p15 (10/05-23)
ASC52 p10 (9/10-22)
ASC52 p9 (9/10-22)
100 KDa
PARP
75 KDa
Non/ specific
20 KDa
Active Caspase3
