# Supplementary material for: Modulation of biological activities in adipose derived stem cells by histone deacetylation
Source: Sci Rep. 2025 Jan 29;15:3629. doi: 10.1038/s41598-024-84652-1 (PMC11779964; doi:10.1038/s41598-024-84652-1)
Supplement: Supplementary file 2 — Supplementary Information 2. [file 41598_2024_84652_MOESM2_ESM.docx]

**Supplementary materials**

Modulation of biological activities in adipose derived stem cells by histone deacetylation

Sallam Abdallah^1^, Mouna Tabebi^1,5^, Sawsan Qanadilo^2^, Neserin Ali^3^, Jing Wang^1^, Pádraig D´Arcy^1^,Wen Zhong^1^, Folke Sjoberg^4^, Moustafa Elmasry ^1,4^, Ahmed El-Serafi ^1,4^

1 The Department of Biomedical and Clinical Sciences (BKV), Linköping University, Linköping, Sweden

2 Department of Biology, The University of Jordan, Amman, Jordan

3 Department of Clinical Sciences, Lund University, Lund, Sweden

4 Department of Hand Surgery and Plastic Surgery and Burns, University Hospital, Linköping, Sweden

5 Clinical Genomics Linköping, Linköping University, Linköping, Sweden


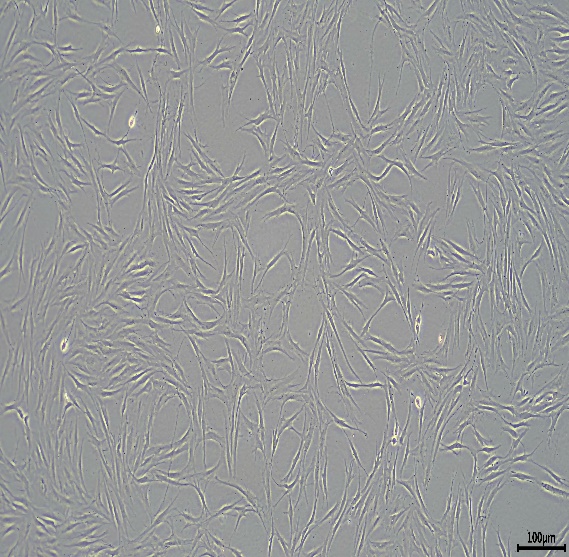


**Untreated**

**EtOH**


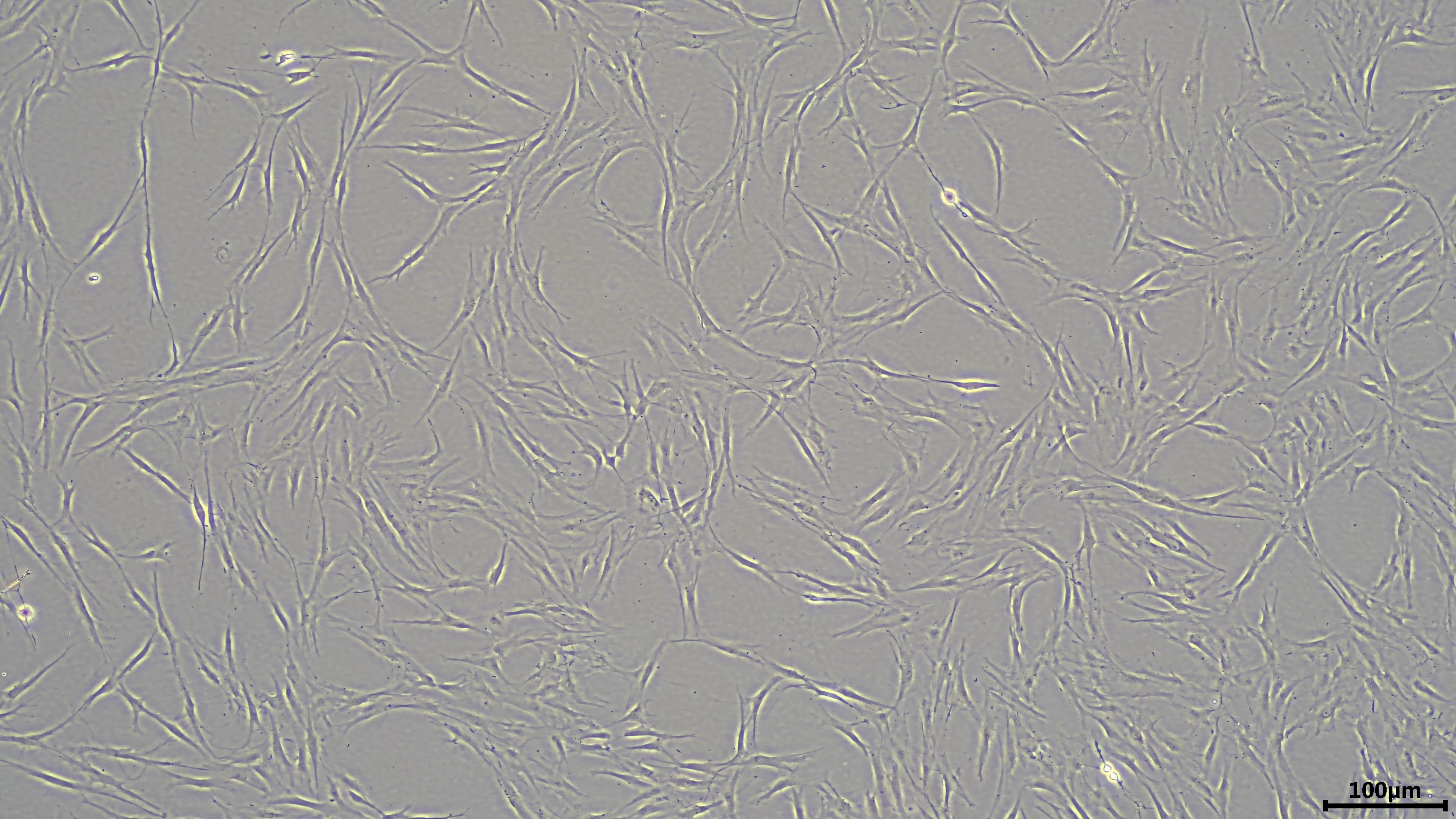


**SAHA**


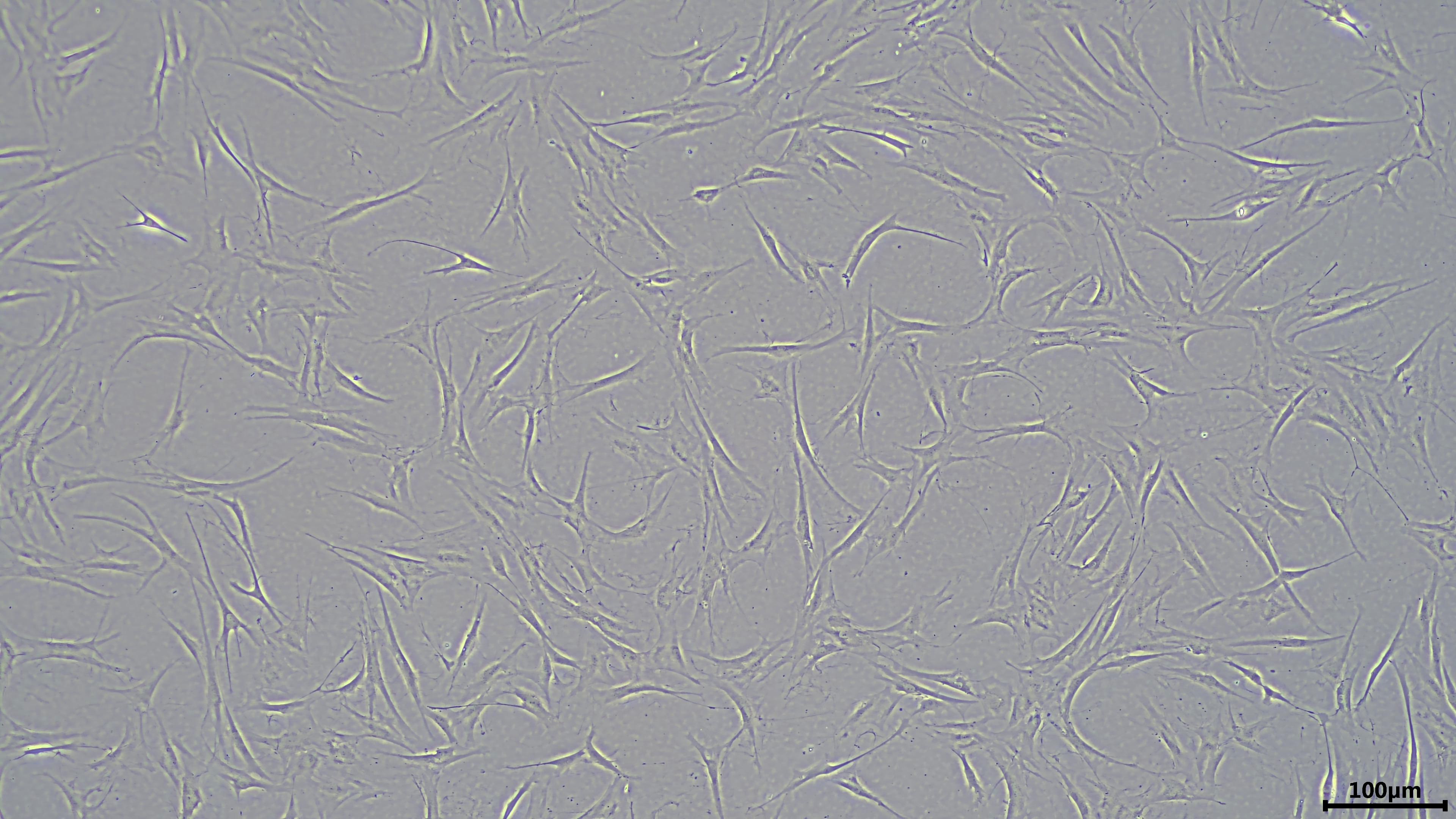


Figure (1): Examination of the morphological properties of ADSCs using the light microscope.

**Microarray results validation:**

To confirm our findings from the microarray analysis, we conducted quantitative real-time PCR. The most significant differentially expressed genes in microarray were chosen for qPCR confirmation of microarray data. The genes are *BGN, ACAN, ASPN, DDAH1, PAPPA* and *IGFBP5* (Table 1). Our qPCR results were in line with the microarray data (Fig 2).

**Table (1): Primers sequences used for the qualitative polymerase chain reaction (q-PCR)**

| Accession number | Gene name | sequence (5' -> 3') | Product length (bp) |  |
| --- | --- | --- | --- | --- |
| NM_001711.6 | Biglycan (BGN) | F 5′-ACACCGGACAGATAGACGTG -3′ | 189 |  |
|  |  | R 5′- CCATCGTCCAGGGTGAAGTC -3′ |  |  |
| NM_001369268.1 | Aggrecan (ACAN) | F 5′-GTGCCTCTCAAGCCCTTGTC-3′ | 152 |  |
|  |  | R 5′-AACAGTGGCCCTGGTACTTG-3′ |  |  |
| NM_017680.6 | Asporin (ASPN) | F 5′-AGGGGTGACGGTGTTCCATA-3′ | 167 |  |
|  |  | R 5′-TTCCTAGGCCCAGCCTTTGTA-3′ |  |  |
| NM_012137.4 | Dimethylarginine dimethylaminohydrolase 1 (DDAH1) | F 5′-CAAAAGGACAAATCAACGAGGTG-3′ | 156 |  |
|  |  | R 5′-TGTGCAGATTCACTAGACCCAA-3′ |  |  |
| NM_002581.5 | Pregnancy-associated plasma protein A, pappalysin 1 (PAPPA) | F 5′-ACAAAGACCCACGCTACTTTTT-3′ | 131 |  |
|  |  | R 5′-CATGAACTGCCCATCATAGGTG-3′ |  |  |
| NM_000599.4 | | Insulin like growth factor binding protein 5 (IGFBP5) | F 5′-AAAGAGCTACCGCGAGCAAG -3′ | 160 |
|  |  |  | R 5′-GCGGTCCTTCTTCACTGCTT -3′ |  |
| NM_001357943.2 | Homo sapiens glyceraldehyde-3-phosphate dehydrogenase (GAPDH) | F 5′- CCTGCACCACCAACTGCTTA-3′ | 120 |  |
|  |  | R 5′-GGCCATCCACAGTCTTCTGAG-3′ |  |  |


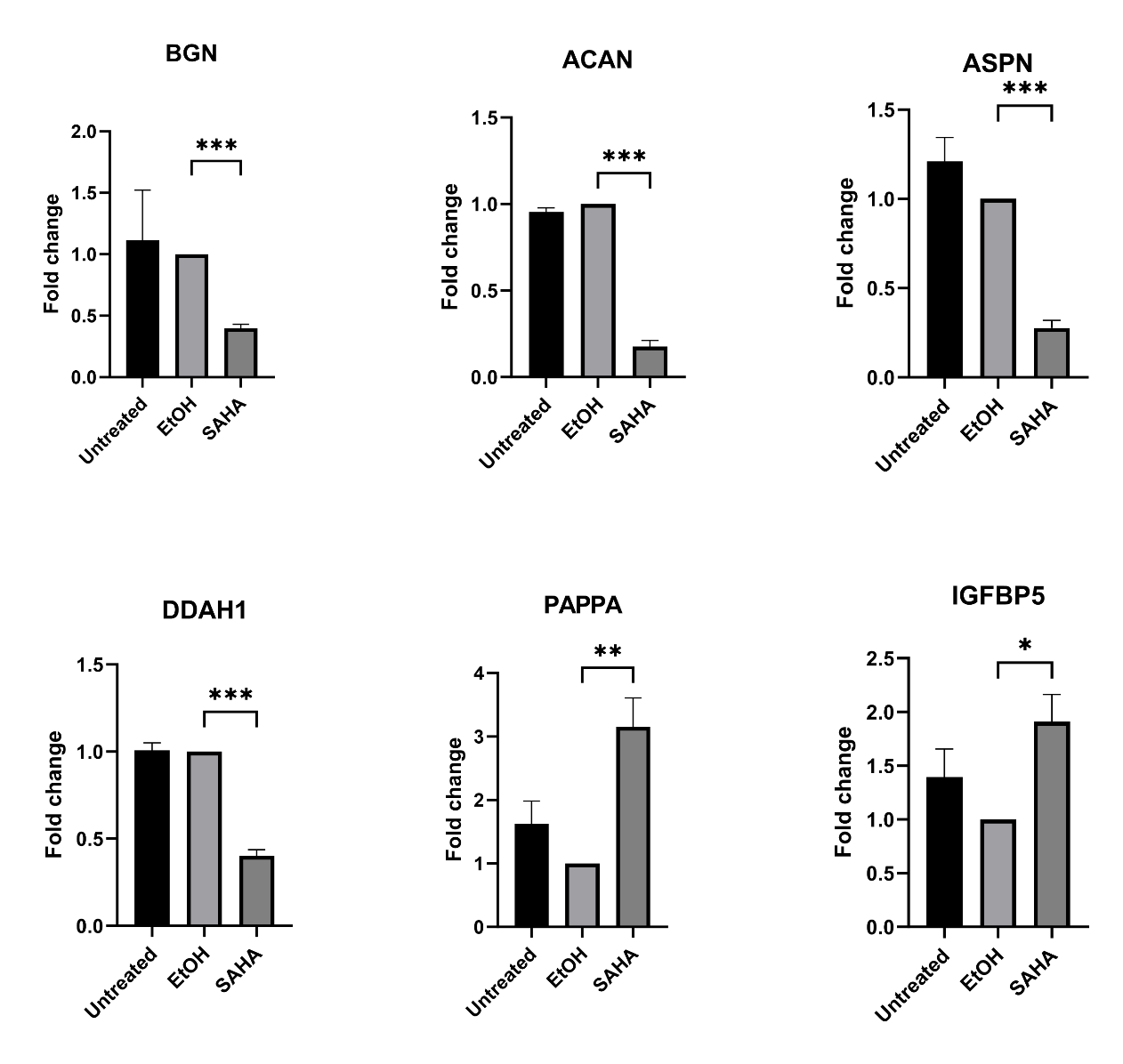


Figure (2): Validation of differentially expressed genes using qPCR for upregulated and down regulated differentially expressed genes using qPCR.

**Microarray data analysis gene ontology (GO) and reactome enrichment pathways.**

**Table (2): GO biological process for the upregulated differentially expressed genes.**

| ID | Description | Gene  Ratio | pvalue | GeneID |
| --- | --- | --- | --- | --- |
| GO:0003170 | heart valve development | 6/70 | 1.4876284790460773e-7 | SMAD6/TGFBR2/ELN/MDM2/PRDM1/SNAI2 |
| GO:0003281 | ventricular septum development | 6/70 | 2.995102097855503e-7 | SMAD6/TGFBR2/MDM2/FZD1/PRDM1/TGFBR3 |
| GO:0003007 | heart morphogenesis | 9/70 | 4.12873973830244e-7 | SMAD6/TGFBR2/ELN/MDM2/ANKRD1/NRG1/FZD1/TGFBR3/SNAI2 |
| GO:0003231 | cardiac ventricle development | 7/70 | 4.3375058898779653e-7 | SMAD6/TGFBR2/MDM2/NRG1/FZD1/PRDM1/TGFBR3 |
| GO:0048732 | gland development | 11/70 | 5.191406140103303e-7 | TGM2/TGFBR2/CCND1/SERPINE2/DKK3/IGFBP5/CAV1/NRG1/TGFBR3/NTN4/SNAI2 |
| GO:0060021 | roof of mouth development | 6/70 | 1.112738246946291e-6 | TGFBR2/FZD1/TIPARP/TGFBR3/OSR1/SNAI2 |
| GO:0003179 | heart valve morphogenesis | 5/70 | 1.8634941521181225e-6 | SMAD6/TGFBR2/ELN/MDM2/SNAI2 |
| GO:0003279 | cardiac septum development | 6/70 | 2.725790304661093e-6 | SMAD6/TGFBR2/MDM2/FZD1/PRDM1/TGFBR3 |
| GO:0003205 | cardiac chamber development | 7/70 | 2.8819890449691583e-6 | SMAD6/TGFBR2/MDM2/NRG1/FZD1/PRDM1/TGFBR3 |

**Table (3): GO biological process for the downregulated differentially expressed genes.**

| ID | Description | Gene Ratio | pvalue | GeneID |
| --- | --- | --- | --- | --- |
| GO:1901342 | Regulation of vasculature development | 6/38 | 6.764219435971e-5 | ENPP2/DDAH1/GLUL/HSPB6/GPNMB/CREB3L1 |
| GO:0045765 | Regulation of angiogenesis | 6/38 | 6.148756431014922e-5 | ENPP2/DDAH1/GLUL/HSPB6/GPNMB/CREB3L1 |
| GO:0000086 | G2/M transition of mitotic cell cycle | 4/38 | 1.78632360896608e-4 | FBXL7/WEE1/CENPF/CALM2 |
| GO:0044839 | Cell cycle G2/M phase transition | 4/38 | 2.637519117160504e-4 | FBXL7/WEE1/CENPF/CALM2 |

**Table (4): GO molecular function for the upregulated differentially expressed genes.**

| ID | Description | Gene Ratio | pvalue | GeneID |
| --- | --- | --- | --- | --- |
| GO:0019838 | Growth factor binding | 8/71 | 6.146784842991506e-8 | SORT1/TGFBR2/IGFBP5/IGFBP3/SRPX2/CD36/RPS19/TGFBR3 |
| GO:0005201 | Extracellular matrix structural constituent | 7/71 | 4.421577213388638e-6 | MXRA5/THSD4/ELN/LUM/SRPX2/MFGE8/EDIL3 |
| GO:0005160 | Transforming growth factor beta receptor binding | 3/71 | 1.0496810094665565e-4 | SMAD6/TGFBR2/TGFBR3 |
| GO:0050431 | Transforming growth factor beta binding | 3/71 | 1.0496810094665565e-4 | TGFBR2/CD36/TGFBR3 |
| GO:0046332 | SMAD binding | 4/71 | 2.335797391582635e-4 | SMAD6/TGFBR2/ANKRD1/TGFBR3 |

**Table (5): GO molecular function for the downregulated differentially expressed genes.**

| ID | Description | Gene Ratio | pvalue | GeneID |
| --- | --- | --- | --- | --- |
| GO:0030021 | extracellular matrix structural constituent conferring compression resistance | 3/39 | 1.3163562251528837e-5 | BGN/ACAN/ASPN |
| GO:0005201 | extracellular matrix structural constituent | 5/39 | 2.9896873907371977e-5 | MFAP4/BGN/ACAN/COL6A3/ASPN |
| GO:0004551 | nucleotide diphosphatase activity | 2/39 | 8.110488119097665e-4 | ENPP2/NUDT4 |

**Table (6): Reactome enrichment pathways analysis for differentially upregulated genes.**

| ID | Description | Gene Ratio | pvalue | GeneID |
| --- | --- | --- | --- | --- |
| R-HSA-381426 | Regulation of Insulin-like Growth Factor (IGF) transport and uptake by Insulin-like Growth Factor Binding Proteins (IGFBPs) | 7/49 | 1.28E-06 | PAPPA/CHRDL1/IGFBP5/IGFBP3/STC2/MFGE8/APLP2 |
| R-HSA-8957275 | Post-translational protein phosphorylation | 6/49 | 8.18E-06 | CHRDL1/IGFBP5/IGFBP3/STC2/MFGE8/APLP2 |

**Table (7): Reactome enrichment pathways analysis for differentially downregulated genes.**

| ID | Description | Gene Ratio | pvalue | GeneID |
| --- | --- | --- | --- | --- |
| R-HSA-3000178 | ECM proteoglycans | 76/10891 | 3.95E-05 | BGN/ACAN/COL6A3/ASPN |
| R-HSA-203615 | eNOS activation | 13/10891 | 4.89E-04 | DDAH1/CALM2 |
| R-HSA-156711 | Polo-like kinase mediated events | 16/10891 | 7.48E-04 | WEE1/CENPF |
| R-HSA-202131 | Metabolism of nitric oxide: NOS3 activation and regulation | 17/10891 | 8.46E-04 | DDAH1/CALM2 |
| R-HSA-1474244 | Extracellular matrix organization | 300/10891 | 8.95E-04 | MFAP4/BGN/ACAN/COL6A3/ASPN |

**Mass spectrometry analysis**

**Gene ontology (GO) and enrichment pathways analysis**

**Table (8): GO biological process for differentially expressed proteins.**

| ID | Description | setSize | pvalue |
| --- | --- | --- | --- |
| GO:0044281 | small molecule metabolic process | 288 | 1E-10 |
| GO:0044255 | cellular lipid metabolic process | 109 | 1.0151E-09 |
| GO:0006091 | generation of precursor metabolites and energy | 91 | 7.4371E-09 |
| GO:0006396 | RNA processing | 130 | 1E-10 |
| GO:0016070 | RNA metabolic process | 349 | 1E-10 |
| GO:0006366 | transcription by RNA polymerase II | 149 | 6.47252E-09 |
| GO:0006357 | regulation of transcription by RNA polymerase II | 141 | 1.19973E-08 |
| GO:0090304 | nucleic acid metabolic process | 406 | 1E-10 |
| GO:0032774 | RNA biosynthetic process | 224 | 1.13626E-08 |
| GO:0006366 | transcription by RNA polymerase II | 149 | 6.47252E-09 |

**Table (9): GO molecular function for differentially expressed proteins.**

| ID | Description | setSize | pvalue |
| --- | --- | --- | --- |
| GO:0001067 | transcription regulatory region nucleic acid binding | 49 | 1.43E-07 |
| GO:0008134 | transcription factor binding | 66 | 5.45E-07 |
| GO:1990837 | sequence-specific double-stranded DNA binding | 55 | 4.73E-08 |
| GO:0000976 | transcription cis-regulatory region binding | 48 | 8.73E-08 |
| GO:0003690 | double-stranded DNA binding | 68 | 1E-10 |
| GO:0140110 | transcription regulator activity | 88 | 2.85E-07 |
| GO:1990837 | sequence-specific double-stranded DNA binding | 55 | 4.73E-08 |
| GO:0003677 | DNA binding | 151 | 1E-10 |
| GO:0003676 | nucleic acid binding | 361 | 1E-10 |
| GO:0016491 | oxidoreductase activity | 139 | 4.65E-09 |

**Table (10): Enrichment pathways analysis for differentially expressed proteins.**

| ID | Description | setSize | pvalue |
| --- | --- | --- | --- |
| hsa00190 | Oxidative phosphorylation | 99 | 1E-10 |
| hsa05208 | Chemical carcinogenesis - reactive oxygen species | 152 | 1E-10 |
| hsa01100 | Metabolic pathways | 768 | 1E-10 |
| hsa05012 | Parkinson disease | 182 | 6.81571E-10 |
| hsa05415 | Diabetic cardiomyopathy | 137 | 8.65371E-08 |
| hsa05020 | Prion disease | 177 | 1.15066E-07 |
| hsa05010 | Alzheimer disease | 230 | 2.224E-07 |
| hsa01200 | Carbon metabolism | 87 | 1.01377E-06 |
| hsa03082 | ATP-dependent chromatin remodeling | 61 | 1.12071E-06 |
| hsa03030 | DNA replication | 30 | 4.26779E-06 |
| hsa04145 | Phagosome | 79 | 5.98443E-05 |
| hsa00020 | Citrate cycle (TCA cycle) | 26 | 8.60637E-05 |


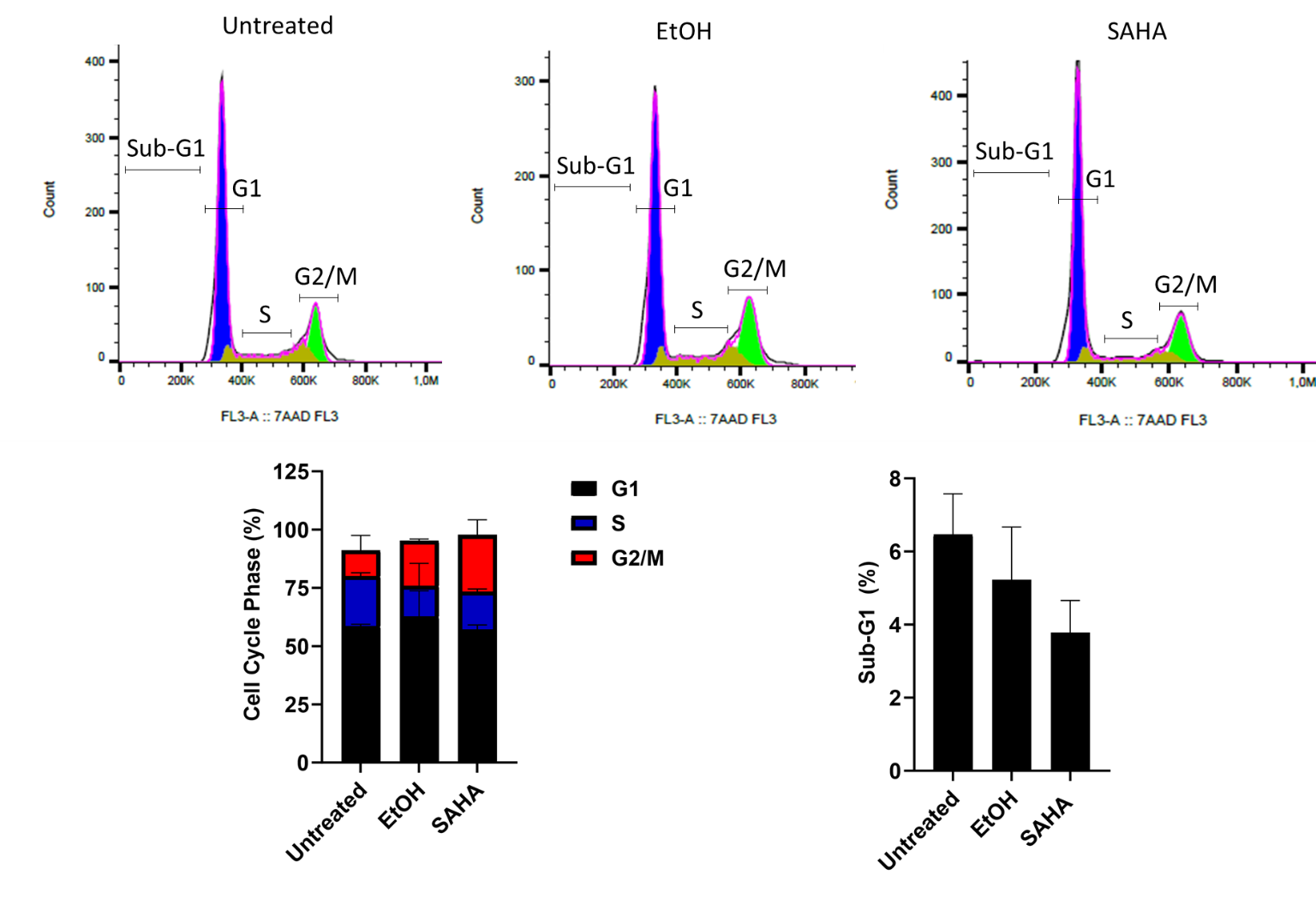


Figure (3) : Cell cycle analysis of ASC52 after being treated with 1000 nM SAHA. Histograms for DNA content along with its quantitative analysis for cell cycle phases and sub-G1 subsets (n=2).


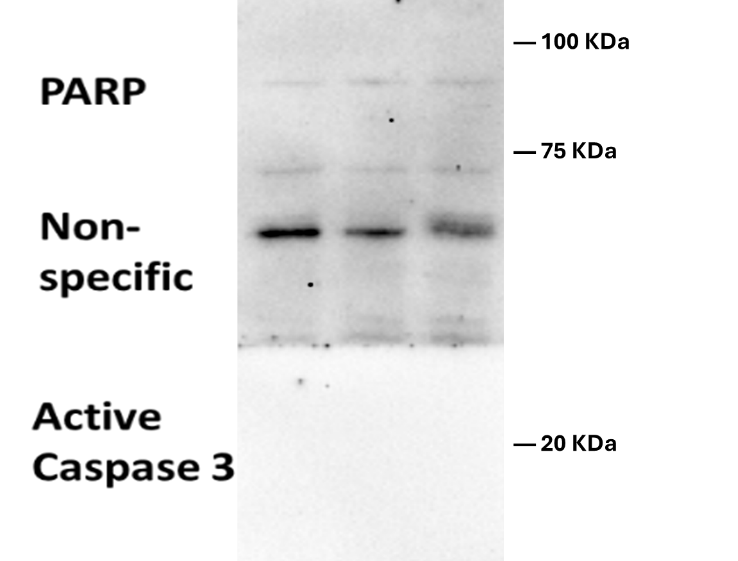


Figure (4): Protein expression of PARP and active caspase-3 using western blotting (n=3).

Full length blots can be accessed in full-length blots file.
